# Children’s and adolescents’ rising animal-source food intakes in 1990–2018 were impacted by age, region, parental education and urbanicity

**DOI:** 10.1038/s43016-023-00731-y

**Published:** 2023-04-20

**Authors:** Victoria Miller, Patrick Webb, Frederick Cudhea, Jianyi Zhang, Julia Reedy, Peilin Shi, Josh Erndt-Marino, Jennifer Coates, Renata Micha, Dariush Mozaffarian, Global Dietary Database

**Affiliations:** 1 Friedman School of Nutrition Science and Policy, Tufts University, Boston, MA, USA.; 2 Department of Medicine, McMaster University, Hamilton, Ontario, Canada.; 3 Population Health Research Institute, Hamilton, Ontario, Canada.; 4 Division of General Internal Medicine and Primary Care, Brigham and Women’s Hospital, Boston, MA, USA.; 5 Department of Food Science and Nutrition, University of Thessaly, Thessaly, Greece.

## Abstract

Animal-source foods (ASF) provide nutrition for children and adolescents’ physical and cognitive development. Here, we use data from the Global Dietary Database and Bayesian hierarchical models to quantify global, regional and national ASF intakes between 1990 and 2018 by age group across 185 countries, representing 93% of the world’s child population. Mean ASF intake was 1.9 servings per day, representing 16% of children consuming at least three daily servings. Intake was similar between boys and girls, but higher among urban children with educated parents. Consumption varied by age from 0.6 at <1 year to 2.5 servings per day at 15–19 years. Between 1990 and 2018, mean ASF intake increased by 0.5 servings per week, with increases in all regions except sub-Saharan Africa. In 2018, total ASF consumption was highest in Russia, Brazil, Mexico and Turkey, and lowest in Uganda, India, Kenya and Bangladesh. These findings can inform policy to address malnutrition through targeted ASF consumption programmes.

Child malnutrition is devastating globally, including micronutrient deficiencies, stunting, underweight and wasting, as well as growing diet-related syndromes, including overweight, obesity and poor metabolic health^1^. While undernutrition decreased over recent decades, an estimated 149 million children under 5 years were stunted in 2020, and 45 million children were wasted^2^. These conditions remain most prevalent in Asia and Africa^2^. In addition, more than 372 million infants and young children have micronutrient deficiencies^3^, which can inhibit healthy growth and development, and increase child mortality^4^; and 38 million children are overweight, with prevalence rates rising steadily in most geographic regions^1,2,5^. The 2021 *Lancet* series on adolescent nutrition emphasized the scarcity of nationally representative data to characterize adolescent diets during this important developmental period^6-8^. Nutrition during childhood and adolescence affects linear growth, body composition, brain growth and development, and immune function^9-11^. The consequences of poor diet during adolescence are exemplified by the doubling of obesity rates, as well as increased anaemia between 1990 and 2016^12^.

Animal-source foods (ASF) have an important dietary influence on the health of children and adolescents. ASF are rich in amino acids, fatty acids and several micronutrients of concern, including iron, zinc, iodine and vitamin A, intake of which is widely deficient in lower-income countries^13^. Specific ASF have other health benefits, such as omega-3 fatty acids in fish, calcium and vitamin D in dairy, probiotics in yogurt and menaquinones in cheese. In a meta-analysis of randomized controlled trials, food-based animal protein supplementation during infancy and early childhood increased child weight, increased height-for-age *z*-score and reduced the risk of stunting, but had no effect on height alone or wasting^14^.

At the same time, different ASF have implications for planetary health–particularly livestock production for red meat, which increases greenhouse gas emissions and has other negative environmental effects^3,15^–as well as negative implications for health later in adulthood, particularly related to processed meat^16^. Thus, beyond direct impacts during childhood and adolescence, food habits and preferences developed at younger ages translate to adulthood^17^, with long-term implications for human and planetary health. Understanding the distribution and heterogeneity in intakes of ASF among children and adolescents around the world is critical for establishing priorities to promote healthy growth and development during childhood, developing dietary preferences to promote health in adulthood and reducing the environmental impacts of food production and consumption.

Some limited nutritional data on ASF consumption are systematically collected, standardized and reported for <5 and ≥15 years of age in low-income countries^18,19^. Prior global or regional reports did not assess ASF intakes among children 5–14 years old^20-22^. In a recent report, we provided global estimates of ASF consumption among children (<19 years)^23^. However, detailed information by age was not reported. Importantly, ASF consumption and malnutrition may also both vary in children and adolescents according to sociodemographic characteristics, such as parental education and urban or rural residence. Furthermore, such variation may not be uniform by world region, country, age group or time. However, distributions of global ASF intake by children and adolescents according to age, parental education and urbanicity have not previously been reported.

To address these major gaps in knowledge, we investigated and report on intakes of major categories of ASF among children and adolescents by age (from birth to age 19 years), sex, parental education level, urbanicity, country and world region between 1990 and 2018, using systematically collected and standardized individual-level national dietary surveys and Bayesian modelling from the Global Dietary Database (GDD).

## Results

### Global, regional and national total ASF consumption

**Fig. 1 ∣:**
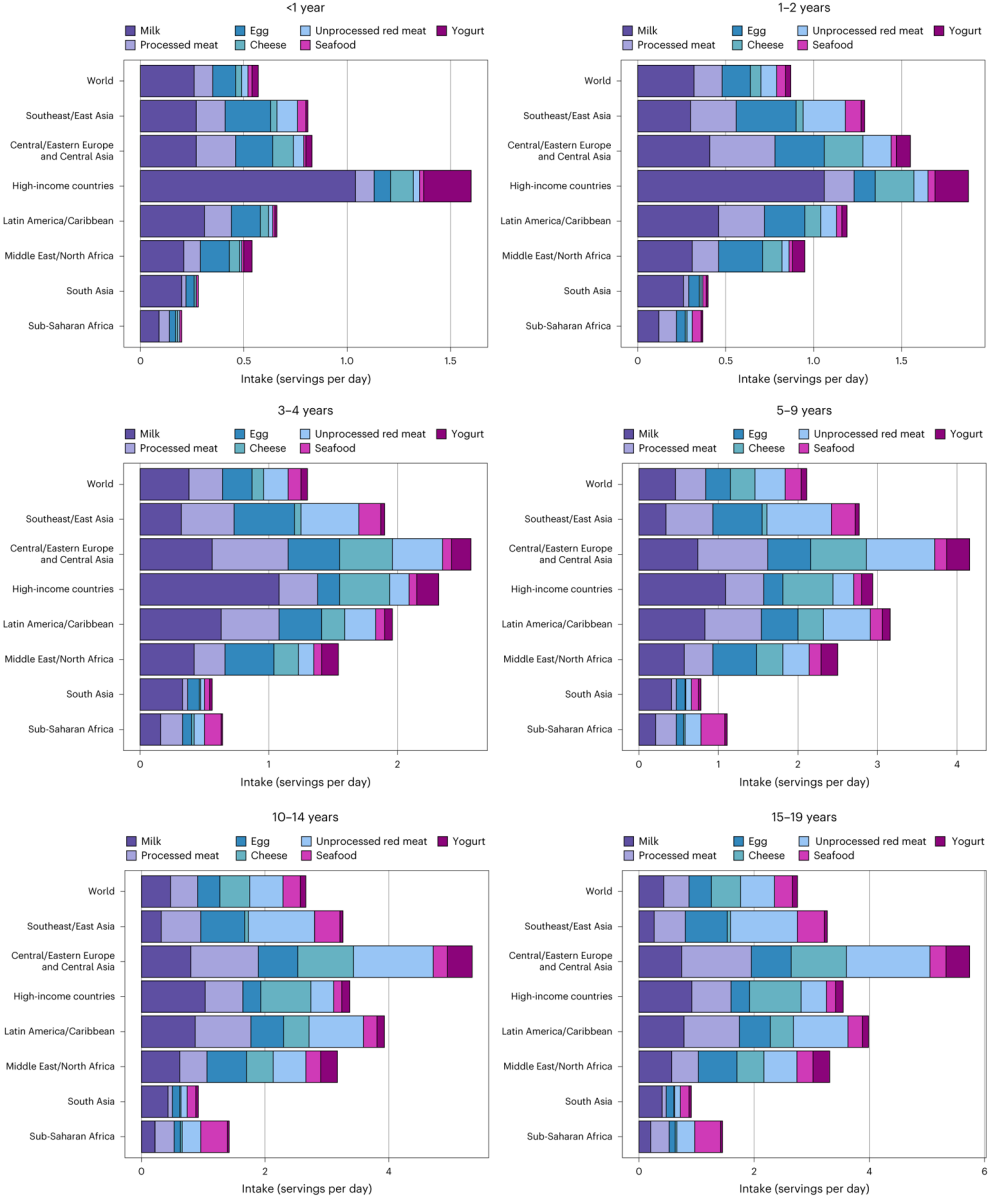
Mean global and regional consumption of ASF in 2018 (servings per day) by age. One serving of unprocessed red meat = 100 g; total processed meat = 50 g; seafood = 100 g; egg = 55 g; cheese = 42 g; yogurt = 245 g; and milk = 245 g.

**Fig. 2 ∣:**
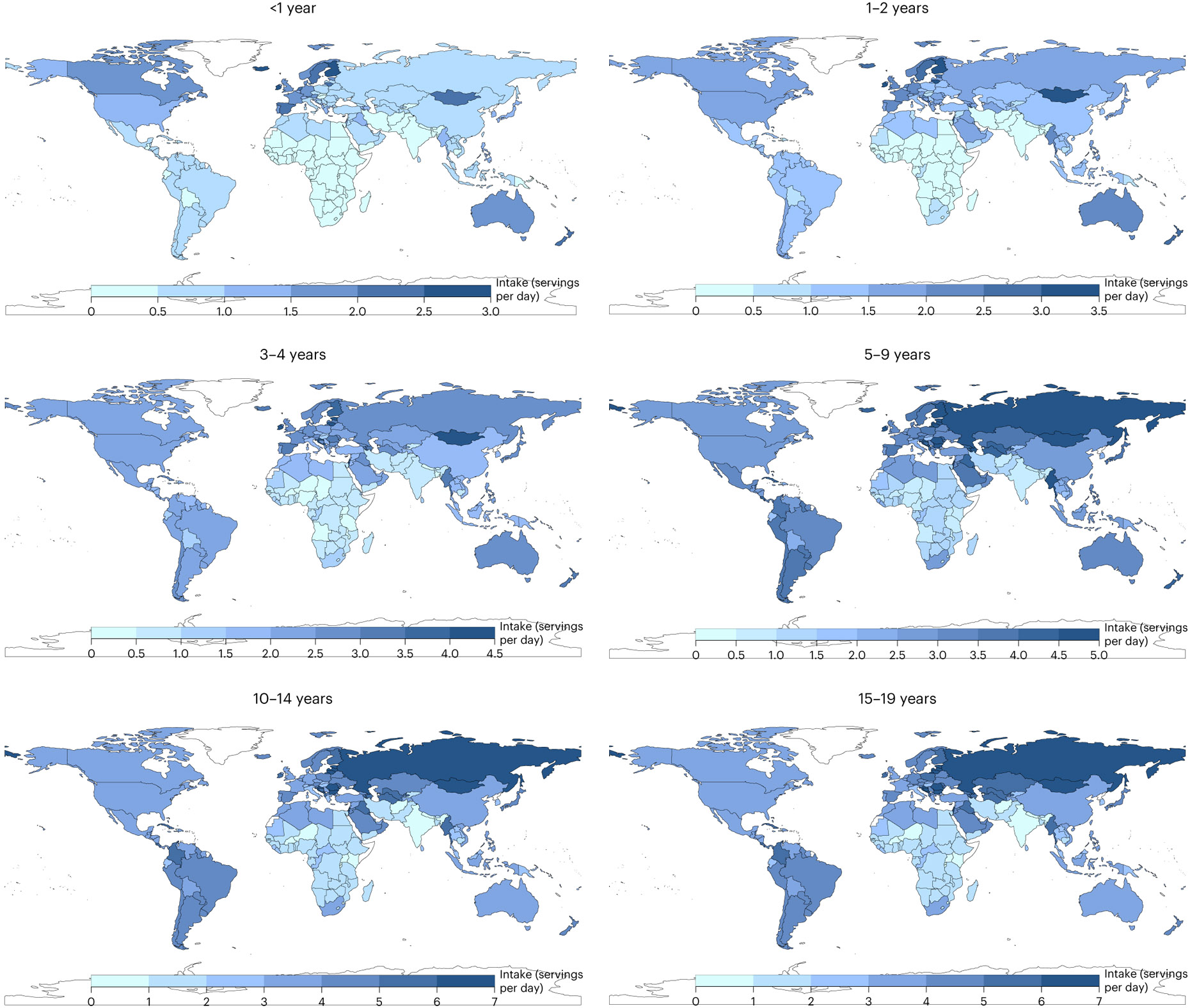
Mean national consumption of total ASF (milk, cheese, yogurt, eggs, seafood, unprocessed red meat and processed meat) in 2018 (servings per day), by age. Of 185 countries, 68, representing 16.3% of the global child population, had mean ASF consumption of ≥3 servings per day.

Worldwide, total ASF consumption by children and adolescents (from birth to age 19 years) in 2018 was 1.9 servings per day (95% uncertainty interval: 1.9, 2.1), ranging from 0.8 in South Asia to 4.2 in Central/Eastern Europe and Central Asia (Fig. 1). Globally, ASF consumption varied 4.5-fold by age group, from 0.6 in age <1 year to 2.5 in 15–19 years. Among the world’s 20 most populous countries, mean ASF intake was highest in Russia, Brazil, Mexico and Turkey (range: 3.0–5.0 servings per day), and lowest in Uganda, India, Kenya and Bangladesh (range: 0.7–0.8; Fig. 2).

### Global, regional and national consumption of dairy

Mean milk intake globally was 103 g d^−1^ (98, 109; equivalent to 0.4 servings per day); cheese intake, 6 g d^−1^ (6, 7; equivalent to 0.1 servings per day); and yogurt intake, 18 g d^−1^ (15, 21; equivalent to 0.5 servings per week; Fig. 1). Globally, mean milk intake was highest among ages 10–14 years (115 g d^−1^; 108, 122), followed by 5–9 years (112 g d^−1^; 105, 118), 15–19 years (105 g d^−1^; 100, 111), 3–4 years (92 g d^−1^; 87, 98), 1–2 years (77 g d^−1^; 73, 82) and <1 year (63 g d^−1^; 58, 67).

A 5.5-fold difference in milk intake was found across regions, from 46 g d^−1^ in sub-Saharan Africa to 252 in high-income countries (Fig. 1). Among populous countries, mean intakes were highest in Mexico, the United States, Brazil and Turkey (222–257 g d^−1^), and lowest in Nigeria, the Democratic Republic of the Congo, Bangladesh and Tanzania (32–43 g d^−1^).

Mean regional cheese intake ranged from 1 g d^−1^ in South Asia and sub-Saharan Africa to 29 in Central/Eastern Europe and Central Asia (Fig. 1). Among populous countries, highest intakes were in the United States, Turkey, Russia and Mexico (15–26 g d^−1^), and lowest in Uganda, the Democratic Republic of the Congo, Bangladesh, India, Tanzania, Pakistan, the Philippines, Nigeria and Kenya (≤1 g d^−1^).

Mean regional consumption of yogurt ranged from 6 to 73 g d^−1^ (Fig. 1). Among populous countries, national intakes were lowest in Uganda, Indonesia, Bangladesh, Tanzania and Kenya (≤5 g d^−1^), and highest in Turkey, Russia, Iran and Mexico (23–105 g d^−1^).

### Global, regional and national consumption of eggs

Mean global intake of eggs was 17 g d^−1^ (15, 20; equivalent to 0.3 servings per day) in 2018, with regional consumption 5- to 7-fold higher in Southeast and East Asia (5–34 g d^−1^), Central/Eastern Europe and Central Asia, the Middle East and Northern Africa, and Latin America and the Caribbean compared with sub-Saharan Africa and South Asia (Fig. 1). Among populous countries, national intakes were lowest in the Democratic Republic of the Congo, Kenya, Tanzania, Uganda and Nigeria (≤4 g d^−1^), and highest in Vietnam, China, Mexico and Russia (33 to 44 g d^−1^). National egg intake was moderately correlated with intake of unprocessed red meat (*r* = 0.6) and processed meat (*r* = 0.5), but not seafood (*r* = −0.03).

### Global, regional and national consumption of seafood

Worldwide, mean seafood consumption was 21 g d^−1^ (20, 23; equivalent to 0.2 servings per day), ranging from 2 g d^−1^ (2, 2) in age <1 year to 31 g d^−1^ (29, 33) in 15–19 years (Fig. 1). Regionally, seafood intake was highest in Southeast and East Asia (32 g d^−1^; 30, 35), followed by sub-Saharan Africa, Central/Eastern Europe and Central Asia, the Middle East and Northern Africa, and Latin America and the Caribbean. Intake was lowest in high-income countries and South Asia (~10 g d^−1^). Among populous countries, mean intakes were highest in Vietnam, Indonesia, the Democratic Republic of the Congo and Bangladesh (37–43 g d^−1^), and lowest in Pakistan, Ethiopia, Turkey, and the United States (≤6 g d^−1^). National seafood intake was negatively correlated with unprocessed red meat intake (*r* = −0.2) and not correlated with processed meat intake (*r* =0.008).

### Global, regional and national consumption of meats

The mean global consumption of unprocessed red meat in 2018 was 40 g d^−1^ (38, 42; equivalent to 0.4 servings per day), varying with age at 3 g d^−1^ (3, 4) in children age <1 year, 9 g d^−1^ (8, 9) in 1–2 years, 19 g d^−1^ (17, 20) in 3–4 years, 38 g d^−1^ (36, 41) in 5–9 years, 54 g d^−1^ (51, 57) in 10–14 years and 59 g d^−1^ (56, 63) in 15–19 years (Fig. 1). By region, unprocessed red meat consumption was highest across all ages in Central/Eastern Europe and Central Asia, and Southeast and East Asia, and lowest in South Asia and sub-Saharan Africa. Among the 20 most populous countries, intakes ranged widely from 3 to 152 g d^−1^: highest in Russia, China, Brazil and Vietnam, and lowest in India, Bangladesh, Ethiopia and Uganda.

Globally, mean consumption of processed meat was 18 g d^−1^ (15, 23; equivalent to 0.4 servings per day), with a 15-fold difference across regions (from 3 g d^−1^ in South Asia to 44 g d^−1^ in Central/Eastern Europe and Central Asia; Fig. 1). Among populous countries, national intakes were highest in the Philippines, Indonesia, Brazil and Russia (40–71 g d^−1^), and lowest in Bangladesh, Kenya, India and Tanzania (≤4 g d^−1^).

Across nations in 2018, unprocessed red meat and processed meat were moderately correlated (*r* = 0.5). In most countries, unprocessed red meat consumption exceeded processed meat consumption; for example, Papua New Guinea (231 versus 10 g d^−1^), Latvia (178 versus 47 g d^−1^), Montenegro (179 versus 52 g d^−1^), Croatia (219 versus 94 g d^−1^) and South Africa (135 versus 19 g d^−1^). Notable exceptions included Sierra Leone (9 versus 67 g d^−1^), Mongolia (58 versus 114 g d^−1^), the Philippines (24 versus 71 g d^−1^), Armenia (52 versus 95 g d^−1^) and Georgia (13 versus 56 g d^−1^).

**Extended Data Fig. 1 ∣:**
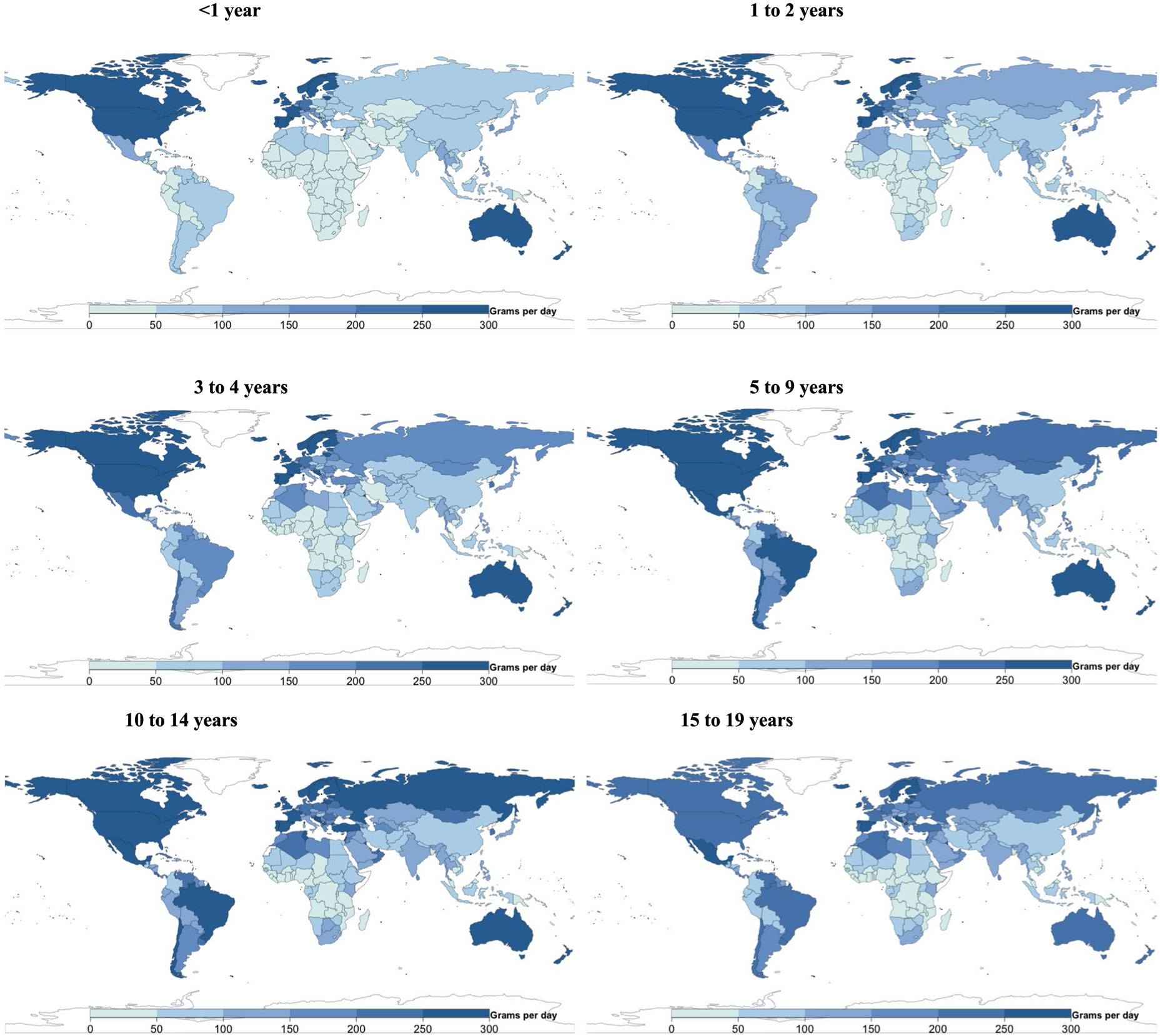
National milk intake (g/d) from 185 countries, by age. Of 185 countries, 20, representing 867 million or 34.0%, had Intakes <1 serving per day. Of 185 countries, 22 had mean intakes of at least one serving of milk (245 g) daily (representing 7.7% of the global child population), and 21 of 185 (representing 10.7% of the global child population) had mean intakes of <1 serving per week.

**Extended Data Fig. 2 ∣:**
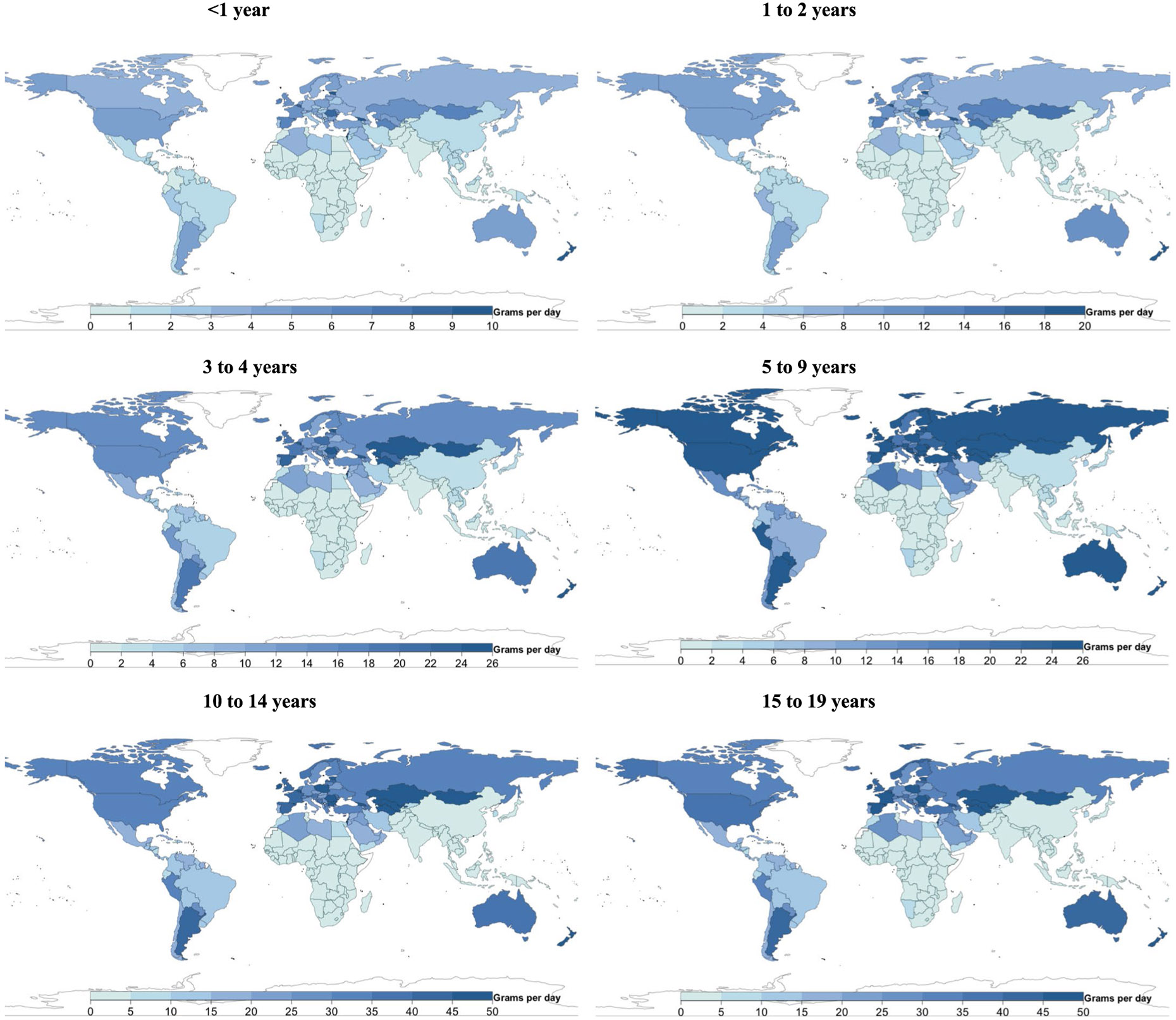
National cheese intake (g/d) from 185 countries, by age. Of 185 countries, 8 had mean intakes of at least one serving of cheese (42 g) daily (representing <1% of the global child population), and 88 of 185 (representing 75.7% of the global child population) had mean intakes of <1 serving per week.

**Extended Data Fig. 3 ∣:**
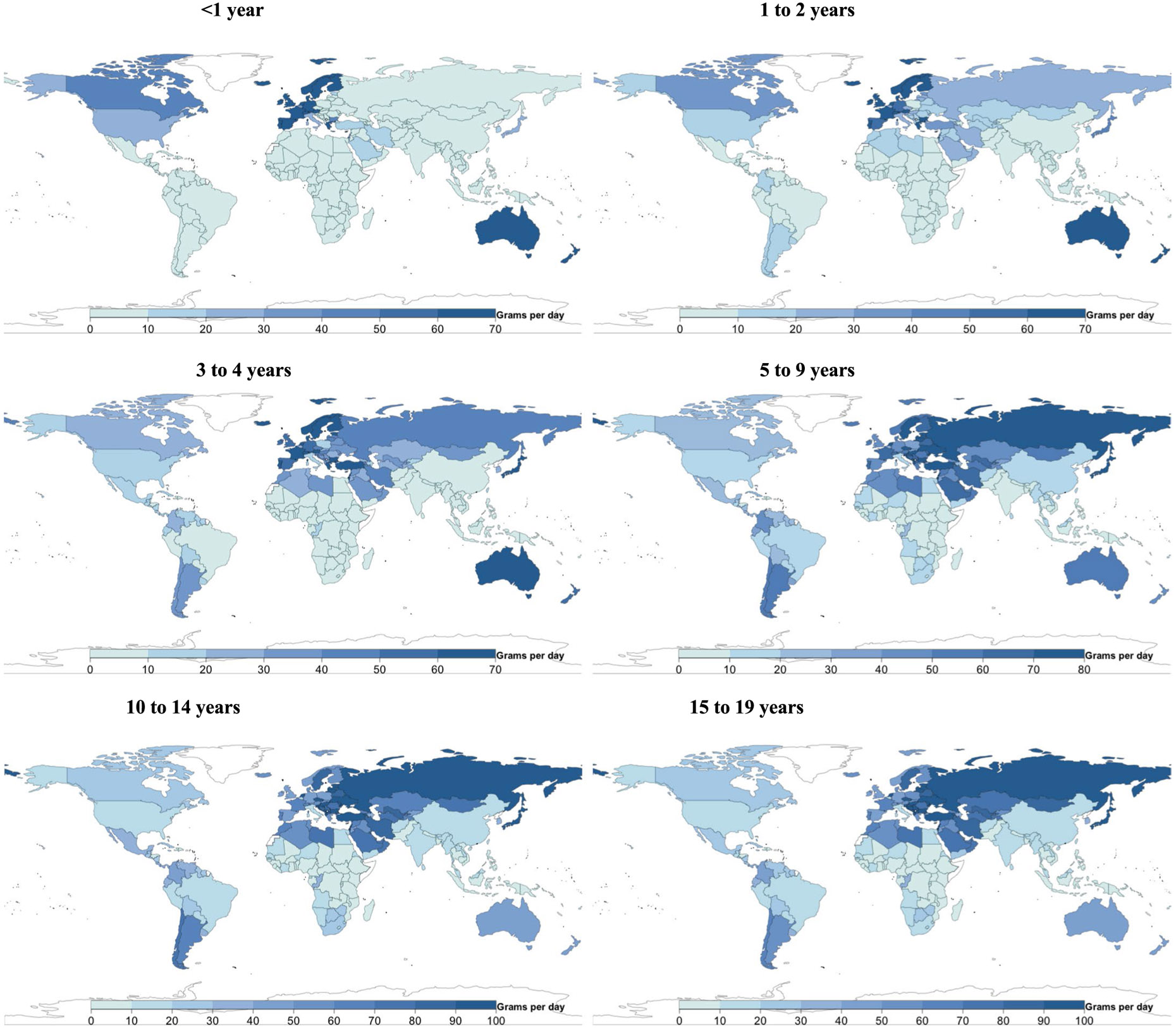
National yogurt intake (g/d) from 185 countries, by age. Of 185 countries, 25 had mean intakes of ≥2 servings of yogurt (245 g) per week (representing 5.2% of the global child population).

**Extended Data Fig. 4 ∣:**
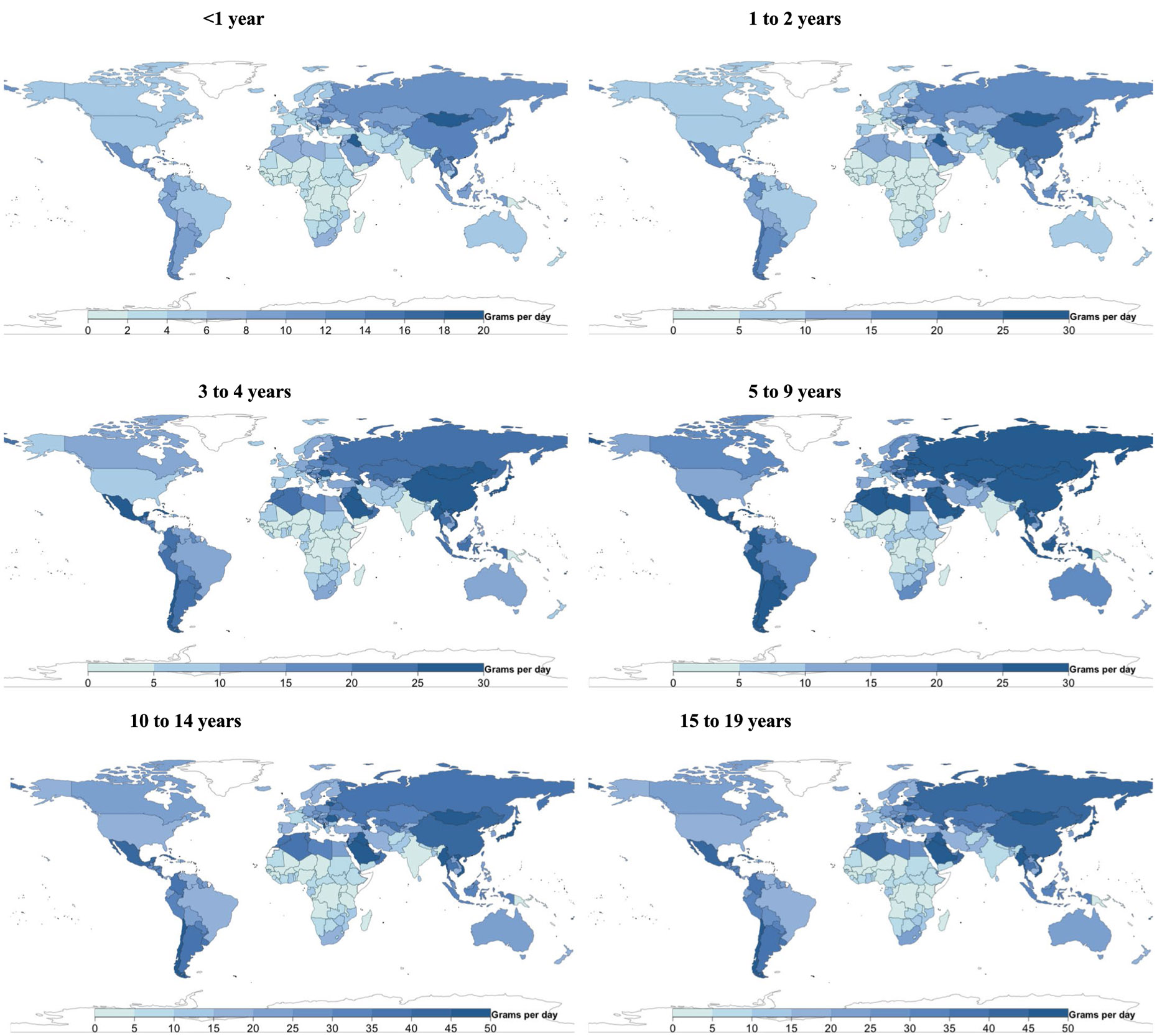
National egg intake (g/d) from 185 countries, by age. Only 5 of 185 countries consumed ≥1 egg (55 g) daily, representing 0.9% of the global child population, and 41 of 185 (representing 41.3% of the global child population) consumed <1 serving per week.

**Extended Data Fig. 5 ∣:**
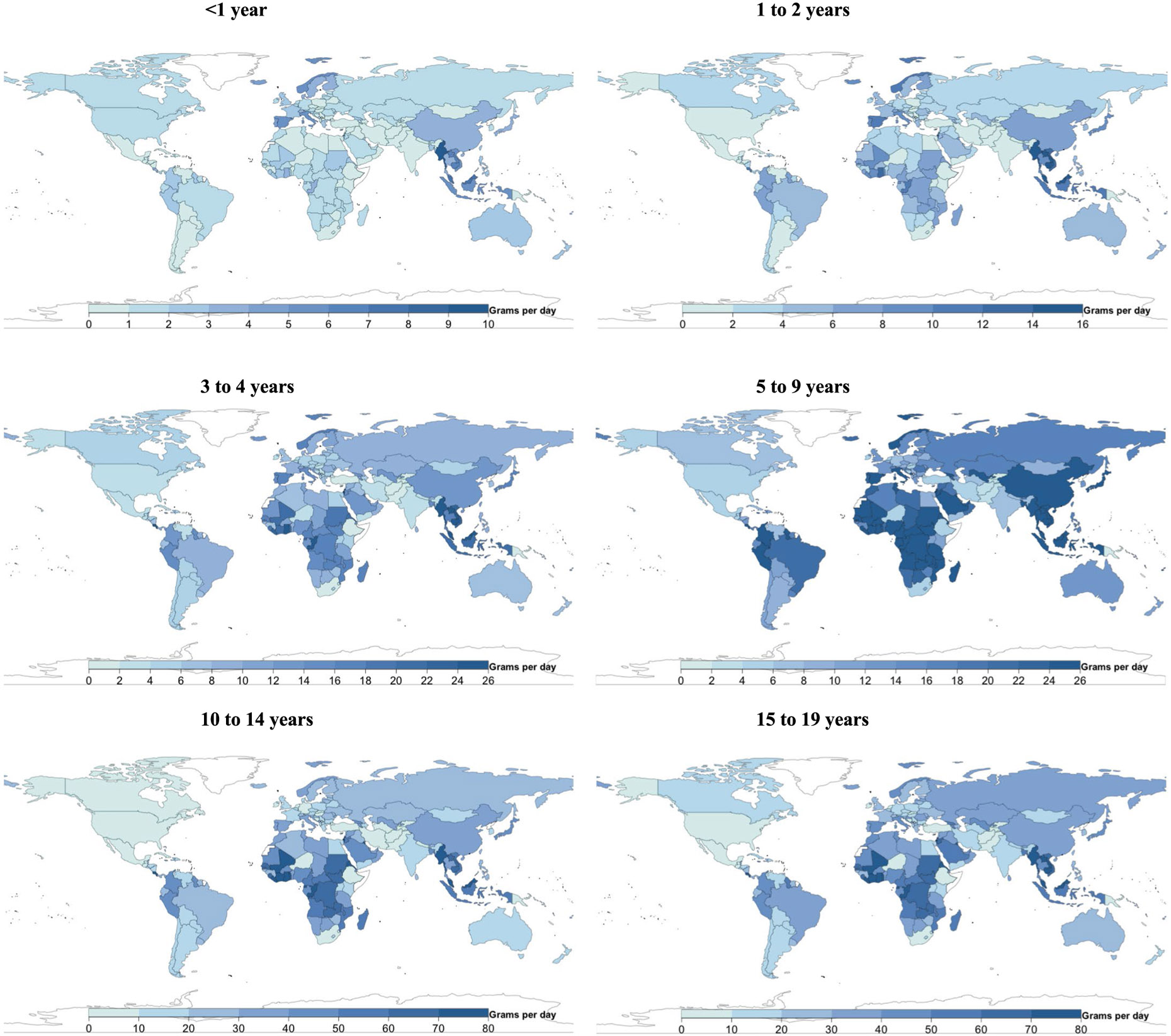
National seafood intake (g/d) from 185 countries, by age. Only 8 of 185 countries consumed a mean of ≥2 servings of seafood (100 g each) per week, representing 2.0% of the global child population, while 56 countries representing 1.2 billion children (45.3% of the global child population) had mean intakes of <1 serving per week.

**Extended Data Fig. 6 ∣:**
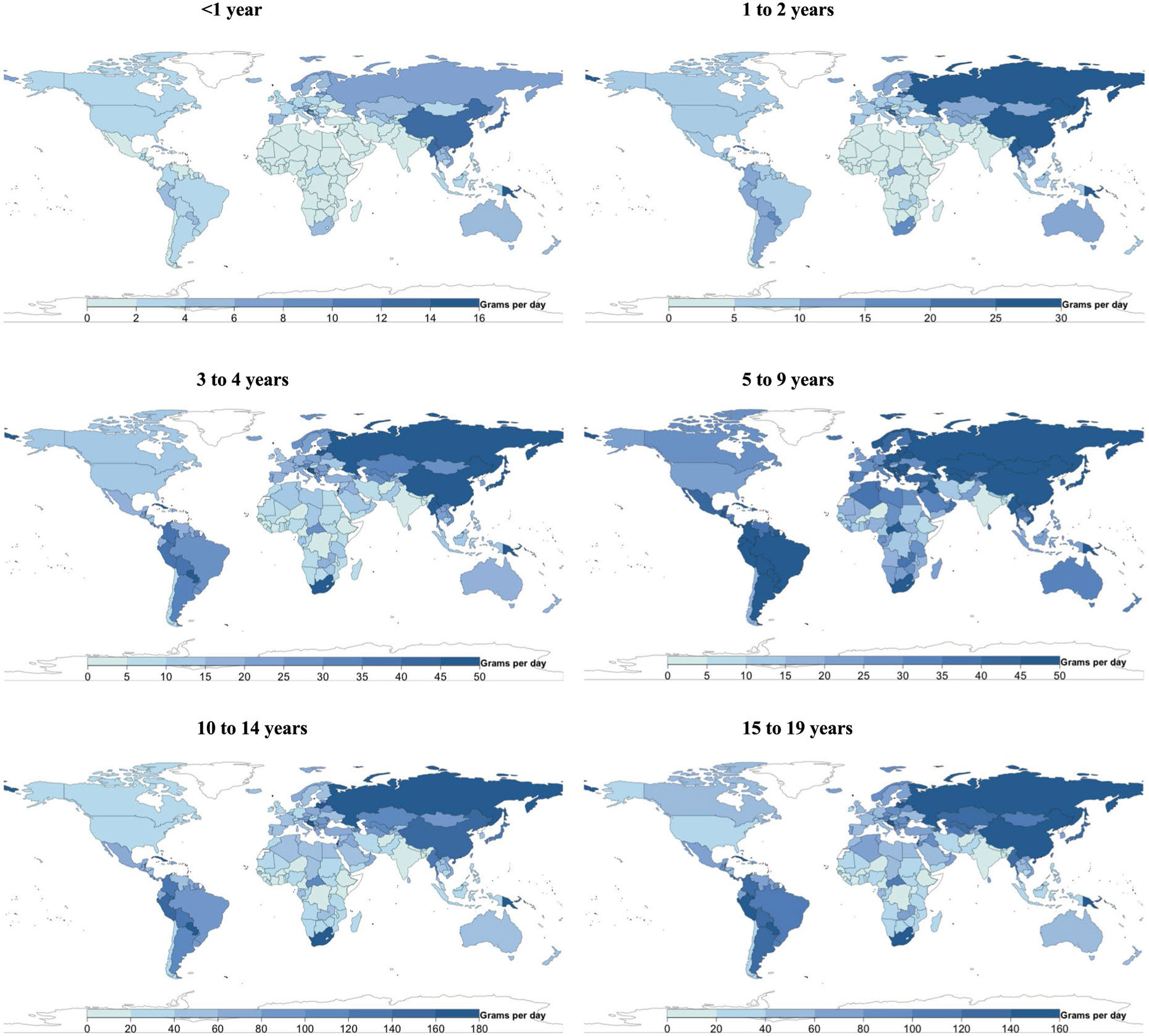
National unprocessed red meat intake (g/d) from 185 countries, by age. Of 185 countries, 14 (representing 17.9% of the global child population) had mean consumption of ≥1 serving of unprocessed red meat (100 g) per day, and 24 of 185 (representing 33.5% of the global child population) had mean consumption of <1 serving per week.

**Extended Data Fig. 7 ∣:**
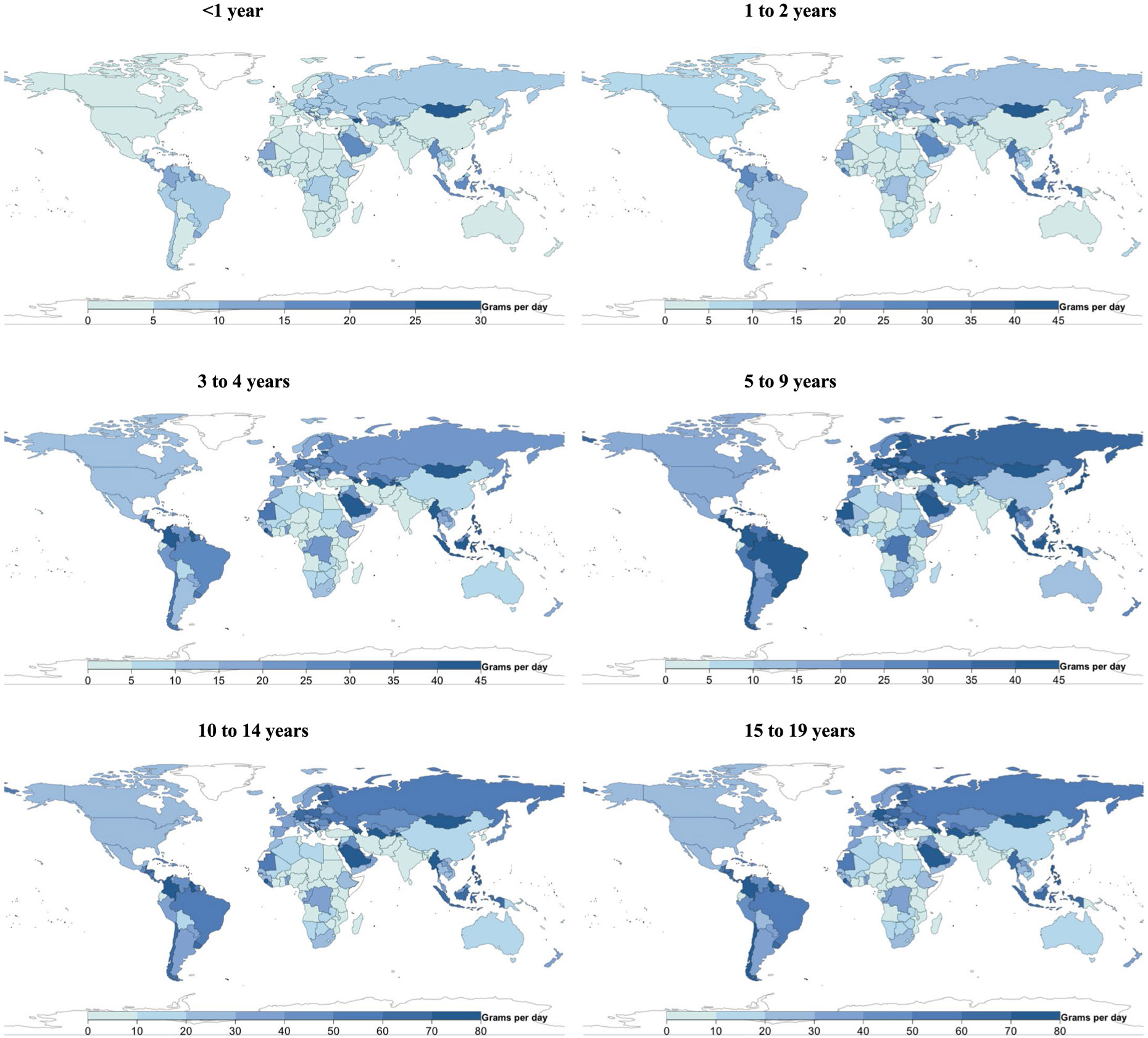
National processed meat intake (g/d) from 185 countries, by age. Of 185 countries, 28 (representing 9.2% of the global child population) had mean intakes of ≥1 serving of processed meat (50 g) daily, and 37 of 185 (representing 44.6% of the global child population) had mean intakes of <1 serving per week.

Extended Data Figs. 1-7 show the consumption of each ASF by age group.

### Differences by sex, education and urbanicity

Globally, mean consumption of most ASF was similar among girls compared with boys, except girls had slightly higher intakes of seafood (+0.04 servings per week; 0.01, 0.07) and milk (+0.03 servings per week; 0.01, 0.06). These differences were generally consistent by age.

**Fig. 3 ∣:**
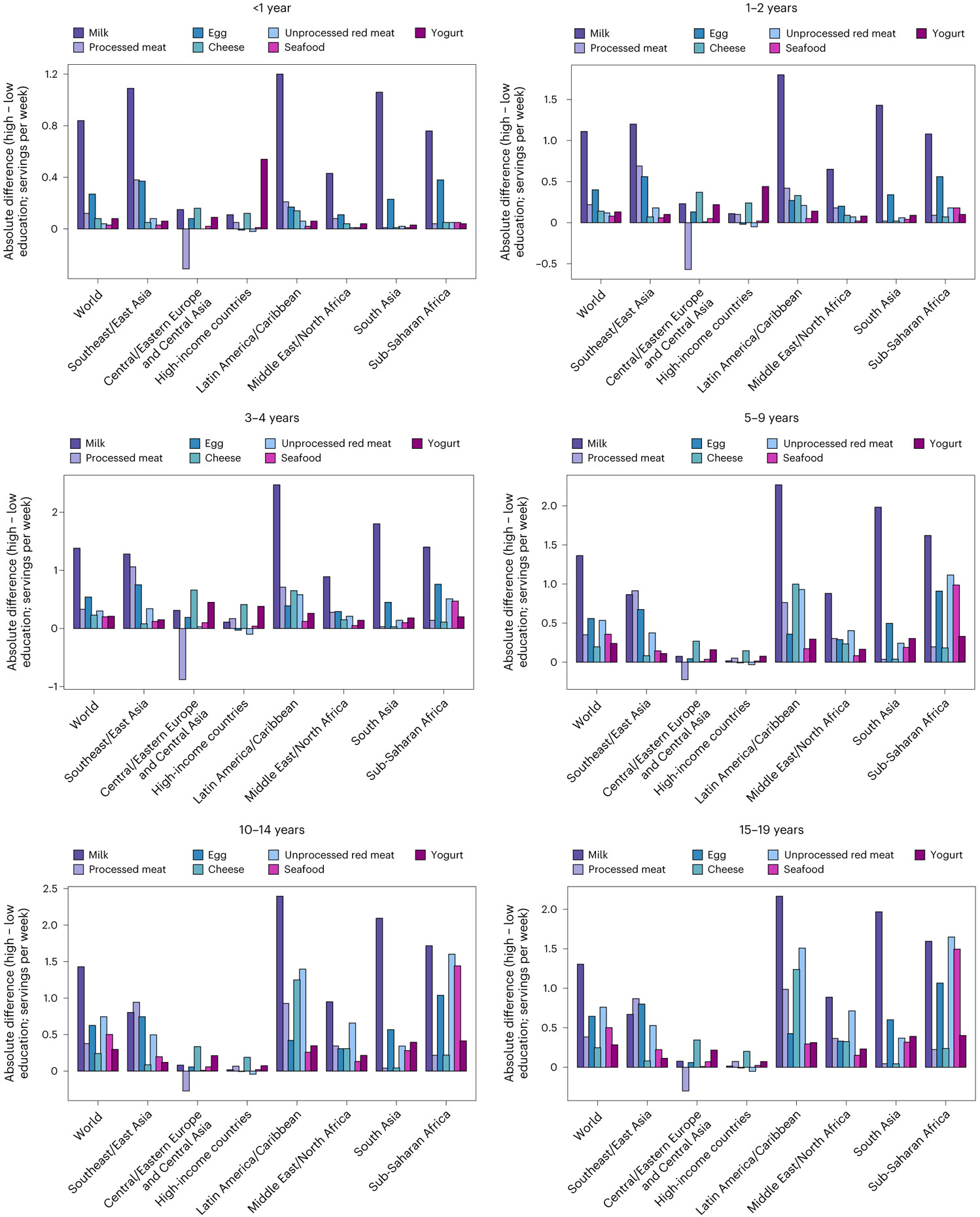
Mean global and regional difference in consumption of ASF according to parental education in 2018, by age. One serving of unprocessed red meat = 100 g; total processed meat = 50 g; seafood = 100 g; egg = 55 g; cheese = 42 g; yogurt = 245 g; and milk = 245 g. The absolute differences by parental education were computed at the stratum level and aggregated to the global and regional mean differences, comparing high education (≥12 years) to low education (<6 years).

Mean intakes of all ASF globally were higher among children and adolescents with more educated parents. In absolute servings, global differences by parental education were largest for milk (+1.7 servings per week; 1.6, 1.9), followed by eggs (+0.7 servings per week; 0.6, 0.9), seafood (+0.5 servings per week; 0.4, 0.6), processed meat (+0.4 servings per week; 0.08, 0.8), yogurt (+0.3 servings per week; 0.3, 0.4), cheese (+0.3 servings per week; 0.2, 0.3) and unprocessed red meat (+0.2 servings per week; 0.2, 0.3; Fig. 3). Among different world regions, the largest differences in absolute intakes by parental education were seen for unprocessed red meat, seafood, eggs and yogurt in sub-Saharan Africa; for milk and cheese in Latin America and the Caribbean; and for processed meat in Southeast and East Asia.

**Fig. 4 ∣:**
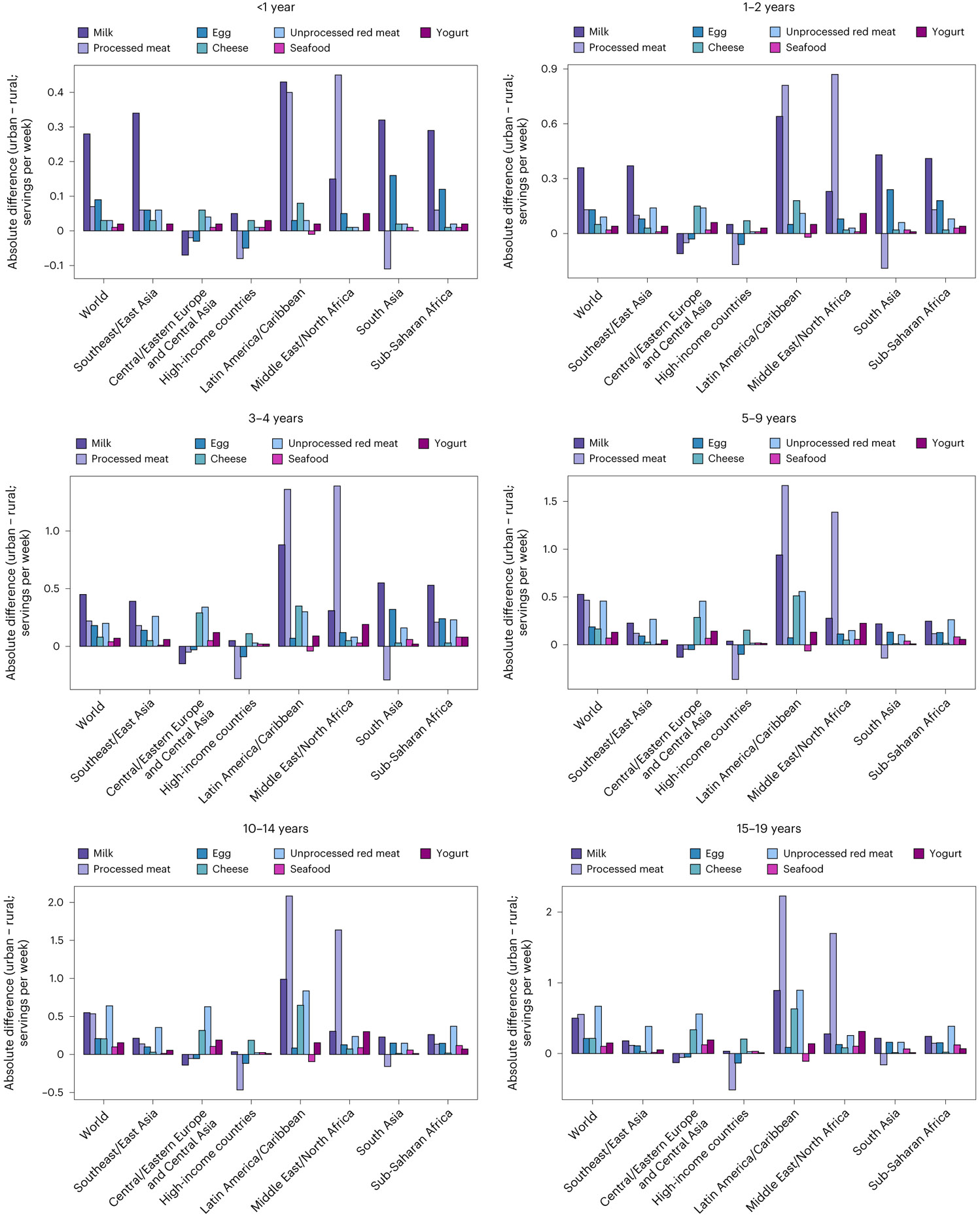
Mean global and regional difference in consumption of ASF between urban residence versus rural residence in 2018 by age. One serving of unprocessed red meat = 100 g; total processed meat = 50 g; seafood = 100 g; egg = 55 g; cheese = 42 g; yogurt = 245 g; and milk = 245 g. The absolute difference by urbanicity was computed as the difference at the stratum level and aggregated to the global and regional mean differences using weighted population proportions.

Globally, children and adolescents residing in urban areas consumed more ASF than those in rural areas. Largest global differences (absolute intakes) in urban versus rural consumption were for processed meat (+0.6 servings per week; 0.06, 1.3) followed by milk (+0.5 servings per week; 0.4, 0.7), eggs (+0.4 servings per week; 0.3, 0.6), yogurt (+0.3 servings per week; 0.2, 0.6), seafood (+0.2 servings per week; 0.09, 0.3), cheese (+0.2 servings per week; 0.02, 0.3) and unprocessed red meat (+0.2 servings per week; 0.1, 0.2; Fig. 4). The corresponding largest regional differences in urban versus rural consumption were for unprocessed red meat, egg, cheese and yogurt in Latin America and the Caribbean; processed meat in Southeast and East Asia; seafood in Central/Eastern Europe and Central Asia, and Latin America and the Caribbean; and milk in high-income countries.

### Differences between 1990 and 2018

**Fig. 5 ∣:**
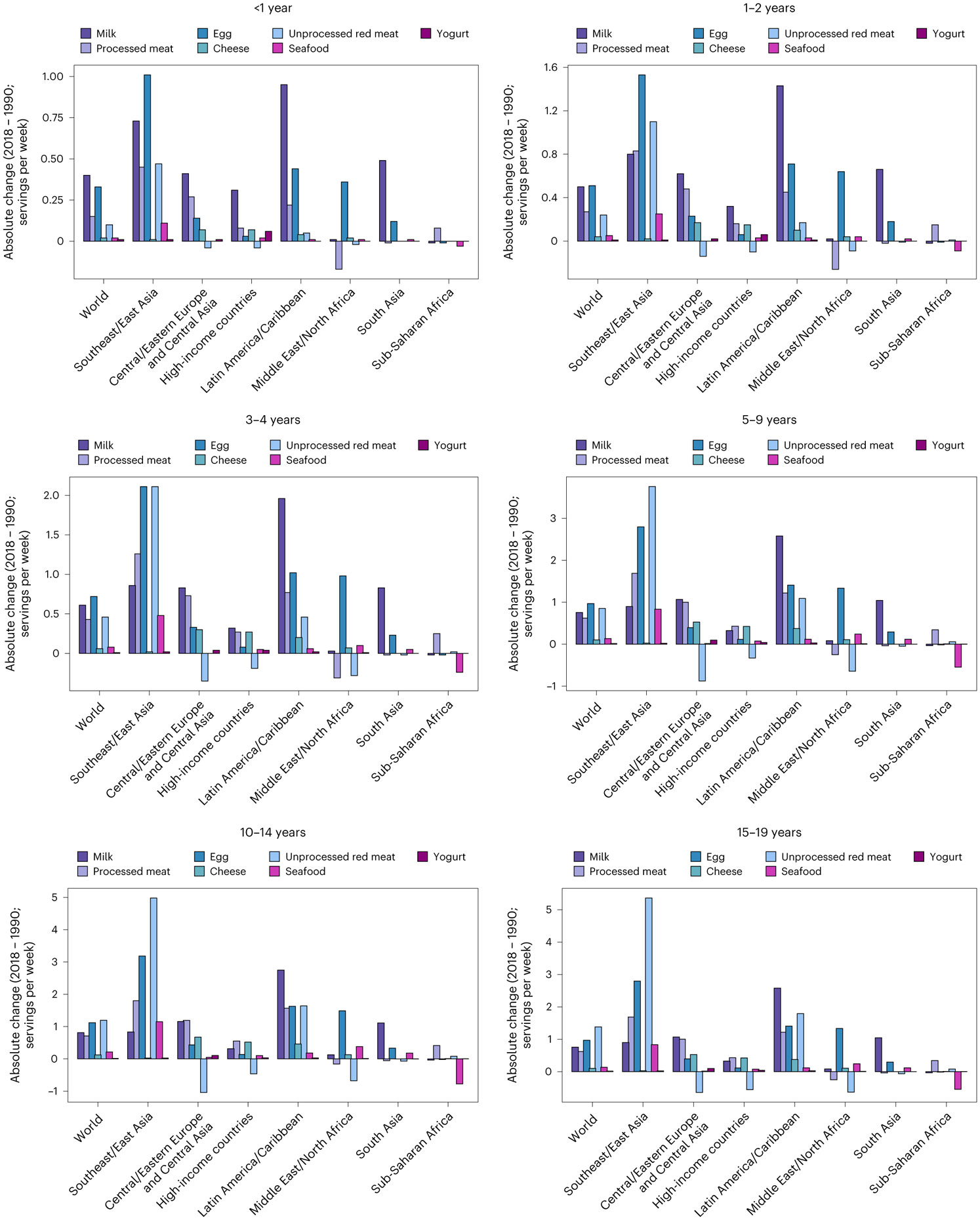
Mean global and regional absolute change in consumption of ASF between 1990 and 2018 (servings per week) by age. 1 serving of unprocessed red meat = 100 g; total processed meat = 50 g; seafood = 100 g; egg = 55 g; cheese = 42 g; yogurt = 245 g; milk = 245 g. The absolute difference between 2018 and 1990 was computed as the difference at the stratum level and aggregated to the global and regional mean differences using weighted population proportions.

**Fig. 6 ∣:**
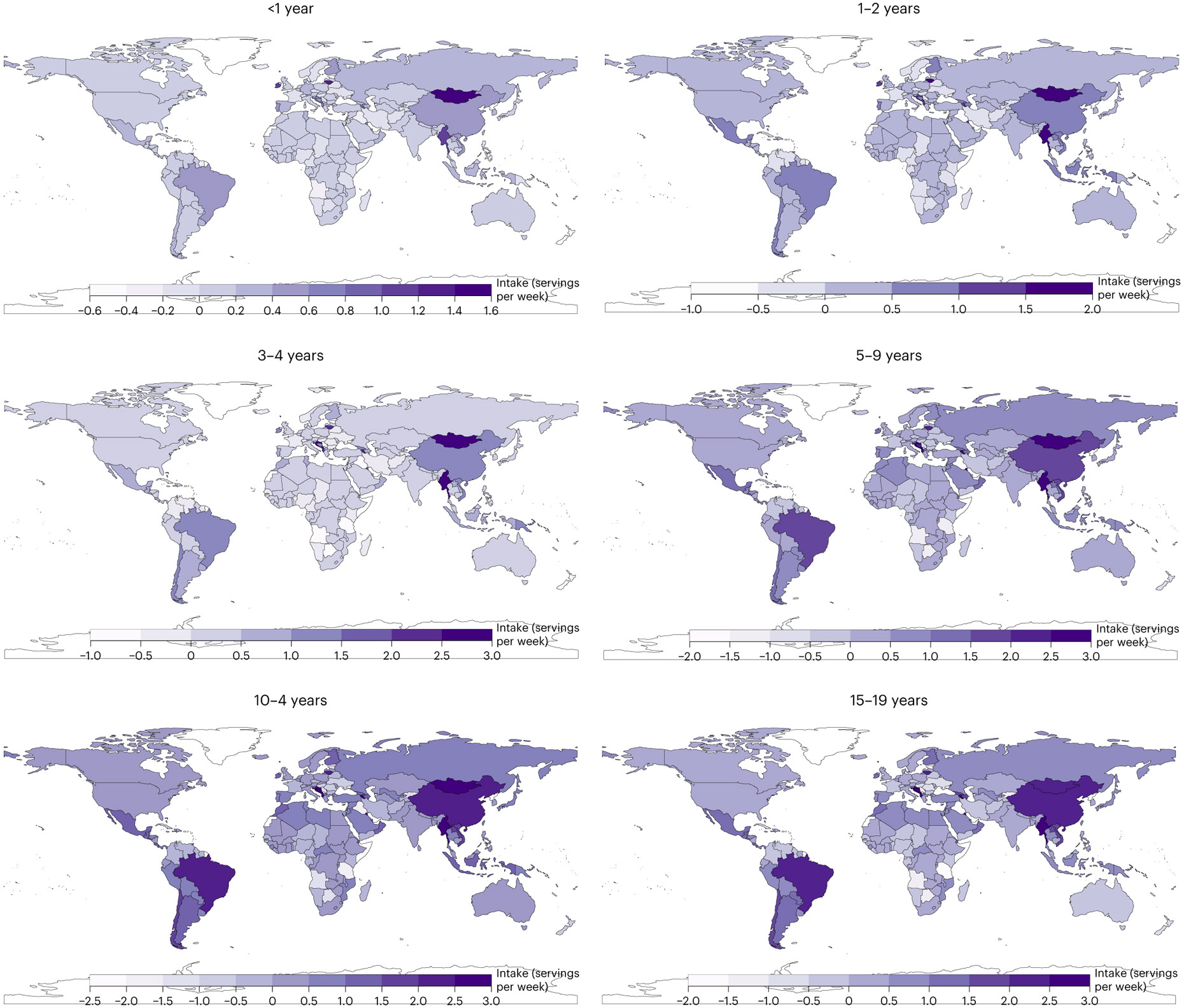
Mean national absolute change in consumption of ASF between 1990 and 2018 (servings per week) by age. The absolute difference between 2018 and 1990 was computed as the difference at the stratum level and aggregated to the global and regional mean differences using weighted population proportions for 2018.

Between 1990 and 2018, mean total ASF consumption increased globally by +0.5 servings per week (0.4, 0.6; Fig. 5). Intake increased in all regions except sub-Saharan Africa, with the largest increase in Southeast and East Asia. Among populous countries, absolute increases were largest in Brazil (+1.8 servings per week; 1.6, 2.0), China (+1.8 servings per week; 1.6, 2.1), Vietnam (+1.6 servings per week; 1.2, 2.1) and Mexico (+1.1 servings per week; 1.0, 1.2). Decreases larger than 0.2 servings were seen in 32 of 185 countries, largest in Tanzania (−1.1 servings per week; −1.3, −0.9), Iran (−0.3 servings per week; −0.4, −0.2) and Kenya (−0.2 servings per week; −0.3, −0.2; Fig. 6). Global increases in total ASF consumption were higher at older ages, with increases of +0.3 servings per week (0.2, 0.3) in children <1 year and +0.7 servings per week (0.6, 0.8) in age 15–19 years.

Regional variation in trends for milk was substantial, from increases of +2.4 servings per week in Latin America and the Caribbean (2.2, 2.6) and +0.3 servings per week in high-income countries (0.3, 0.4), to no change in the Middle East and Northern Africa, and decreases in sub-Saharan Africa (−0.03 servings per week; −0.06, −0.01; Fig. 5). Among populous countries, Brazil experienced the largest increase (+4.5 servings per week; 4.0, 5.2), followed by Mexico (+4.1 servings per week; 3.7, 4.5), Turkey (+2.3 servings per week; 1.5, 3.6) and Russia (+2.3 servings per week; 1.7, 3.0), while the largest decreases were in the Philippines (−2.7 servings per week; −3.0, −2.4), Iran (−1.7 servings per week; −1.9, −1.5) and Kenya (−0.9 servings per week; −1.0, −0.8).

Cheese intake increased globally by +0.1 servings per week (0.05, 0.2), with increased consumption in the high-income countries (+0.4 servings per week; 0.2, 0.7), and Latin America and the Caribbean (+0.4 servings per week; 0.2, 0.5; Fig. 5). Among populous nations, largest national increases were in Mexico (+0.6 servings per week; 0.5, 0.7), the United States (+0.4 servings per week; 0.3, 0.5), Iran (+0.3 servings per week; 0.2, 0.4) and Brazil (+0.2 servings per week; 0.1, 0.3); only Turkey experienced a decrease (−0.4 servings per week; −0.5, −0.3; Fig. 6).

Global yogurt consumption was stable between 1990 and 2018 (+0.02 servings per week; −0.01, 0.04; Fig. 5). Intakes did not substantially increase or decrease in any region. Among populous nations, intakes increased in Mexico (+0.04 servings per week; 0.02, 0.07), the United States (+0.01 servings per week; 0.01, 0.02) and Egypt (+0.01 servings per week; 0.01, 0.03). A decrease in intake occurred in Iran (−0.2 servings per week; −0.3, −0.07), Turkey (−0.04 servings per week; −0.08, −0.01) and Brazil (−0.03 servings per week; −0.06, −0.01).

Egg consumption doubled globally, increasing by +1.0 servings per week (0.8, 1.2), with increases in all regions (range +0.3 to +2.8) except high-income countries and sub-Saharan Africa, largest in Southeast and East Asia (Fig. 5). Across populous countries, greatest increases were in Vietnam (+4.8 servings per week; 3.4, 6.6), China (+3.7 servings per week; 2.5, 5.2) and Mexico (+2.5 servings per week; 2.3, 2.8). Greatest decreases were in Ethiopia (−0.5 servings per week; −0.6, −0.4), Tanzania (−0.4 servings per week; −0.5, −0.3), Kenya (−0.4 servings per week; −0.4, −0.3) and the Democratic Republic of the Congo (−0.1 servings per week; −0.1, −0.09).

Globally, seafood intake increased by +0.2 servings per week (0.1, 0.2), a small change but still representing a near doubling between 1990 and 2018 (Fig. 5). The largest regional increase was +0.9 servings per week (0.8, 1.1) in Southeast and East Asia, and the largest decrease was −0.5 servings per week (−0.6, −0.4) in sub-Saharan Africa. Across populous countries, greatest increases were in Vietnam (+2.3 servings per week; 1.6, 3.3), China (+1.2 servings per week; 1.0, 1.3), Bangladesh (+0.9 servings per week; 0.7, 1.1) and Indonesia (+0.8 servings per week; 0.7, 1.0). Largest decreases were in Tanzania (−7.6 servings per week; −9.1, −6.3), the Philippines (−2.4 servings per week; −2.7, −2.2), Uganda (−0.9 servings per week; −1.0, −0.8) and the Democratic Republic of the Congo (−0.5 servings per week; −0.6, −0.4).

Notably, unprocessed red meat intake only meaningfully increased in Southeast and East Asia (+3.9 servings per week; 3.5, 4.4) and Latin America and the Caribbean (+1.2 servings per week; 1.1, 1.3), while it declined in Central/Eastern Europe and Central Asia (−0.7 servings per week; −1.0, −0.5; Fig. 5). Among populous countries, largest increases were in China (+6.1 servings per week; 5.3, 6.9), Brazil (+2.4 servings per week; 2.2, 2.7), Mexico (+1.1 servings per week; 1.0, 1.3) and Egypt (+1.0 servings per week; 0.9, 1.1), and largest decreases were in Russia (−1.5 servings per week; −1.8, −1.0), Iran (−1.0 servings per week; −1.2, −0.9) and the United States (−0.4 servings per week; −0.4, −0.3).

Increases in processed meat consumption occurred in 4 regions (range +0.5 to +1.5 servings per week), with no change in the Middle East and Northern Africa, South Asia or sub-Saharan Africa (Fig. 5). Among populous countries, increases were seen in 11 of 25 nations, largest in the Philippines (+6.2 servings per week; 4.9, 7.7), Brazil (+3.9 servings per week; 2.9, 5.1) and Indonesia (+3.7 servings per week; 1.4, 6.3); and a decrease only in Mexico (−0.7 servings per week; −1.0, −0.4).

## Discussion

We systematically quantified ASF consumption among children and adolescents in 185 countries in 1990 and in 2018. Our results show that global mean intake of total ASF was almost 2 servings per day in 2018 but varied from <1 serving per day in South Asia to >4 servings per day in Central/Eastern Europe and Central Asia. Our findings also identified substantial heterogeneity by type of ASF, age, parental education and urban versus rural residence. As ASF intake is important for both human and planetary health, with differing impacts by life stage and type of ASF, these findings are highly relevant to health and nutrition professionals, scientists, policymakers, the private sector and the public, and can be used to inform policies and programmes to improve ASF consumption among specific population groups.

At global and regional levels, mean total ASF intake was lower at younger ages. Generally, milk intake contributed around half of total ASF servings in the youngest age groups, while intakes of ASF were more varied in older age groups. Consumption of cheese, seafood and especially yogurt generally contributed the fewest daily servings across all ages and regions. These findings demonstrate consistent shifts in the types and quantities of ASF consumed as children and adolescents age across the globe. These results also support the importance of early life interventions to improve types of ASF consumption for optimal childhood health, dietary preferences for disease prevention in adulthood and planetary health targets.

Infants and young children are particularly vulnerable to various forms of undernutrition due to their high energy and nutrient needs, small stomachs and increased sensitivity to anti-nutrients^24,25^. In addition to obvious manifestations such as stunting, poor diet during early life may adversely affect metabolic health, cognitive performance, physical activity and immune function, creating negative health and economic consequences in adulthood^13^. The prevalence of micronutrient deficiencies including for iron, zinc, iodine, folate, vitamin A, vitamin B12 and vitamin D are high globally, but disproportionately impact children in South Asia and sub-Saharan Africa^26-28^, where intakes of ASF were lowest. Organ meats, fish, eggs and ruminant meat are micronutrient dense and can help address nutritional gaps among children in low-income countries^29-31^. Yet, we found that intakes of seafood, eggs and unprocessed red meat were very low among children in South Asia and sub-Saharan Africa, and increased only slightly over time, highlighting the urgency of new dietary policies to address childhood and adolescent malnutrition in these regions.

Children and adolescents’ intakes of ASF were highest in Central/Eastern Europe and Central Asia, Latin America and the Caribbean, and high-income countries. In adulthood, different ASF subtypes have varying associations with diet-related chronic diseases, with beneficial associations observed for fish, milk, cheese and yogurt, generally neutral associations for eggs, and harmful associations for red meat and especially processed meat^16^. Additionally, compared with ruminant meats (goat, lamb/mutton and especially beef), eggs, dairy and seafood have lower environmental impacts, including for greenhouse gas emissions, land use, energy use, acidification potential and eutrophication potential^32-34^. Yet, our results show that consumption of red and processed meat was generally higher than other ASF in these regions, especially among adolescents. Among populations with adequate ASF consumption, substituting seafood and dairy in place of meats would engender both human and planetary health benefits^35^.

Several studies have demonstrated nutritional benefits from regularly consuming ASF during childhood^25,36,37^, but mean ASF consumption remains less than 2 servings per day, particularly in low-income countries. Several factors may be a barrier to ASF intake, including affordability, nutritional knowledge, parental education, household income, household ownership of livestock and social norms and beliefs^25,38,39^. Our findings show higher ASF consumption among children and adolescents with more educated parents, and children and adolescents residing in urban areas. Similarly, the International Study of Childhood Obesity, Lifestyle and the Environment study reported that household socioeconomic status was related to children’s dietary intakes across countries^40^. Interestingly, we found that differences in ASF intake by both parental education and urbanicity tended to be larger at older ages for all ASF subtypes. Considering our findings, additional research examining the influence of parental education and urbanicity on diet across childhood and adolescence is needed within and across countries.

Previously, a study of 130,432 children aged 6–23 months enrolled in the Demographic Health Survey found that 50.7% of children 6–11 months, 66.4% 12–17 months and 69.8% 18–23 months consumed at least 1 ASF per day^25^. Among this population, milk and red/white meat were more commonly consumed than eggs or fish^25^, which generally agrees with our results. Data from 41 countries collected by the Gallup World Poll reported that most adolescents (≥15 years of age) consumed at least 1 ASF per day, but with less than 70% of adolescents from Mozambique, Burkina Faso and Tanzania consuming 1 ASF per day^41^. Consistent with our findings, the percentage of adolescents consuming at least 1 daily serving of unprocessed red meat was highest in Vietnam and China^41^.

Strengths of our study should be highlighted. Bayesian hierarchical modelling was used to incorporate data on >400 individual-level surveys and address heterogeneity, and sampling and modelling uncertainty^23^. We estimated intakes of several ASF subtypes, many of which have not previously been systematically reported for children >5 years of age. We investigated global, regional and national differences by important demographic characteristics, and over time.

Potential limitations should also be considered. Despite extensive efforts to identify data on ASF intakes, survey availability was limited for some ASF subtypes (for example, cheese and yogurt), age groups, countries and years^23,42^. Differences in individual survey design and dietary assessment required certain decisions about serving sizes, food group definitions, energy adjustment and the disaggregation of household-level data when standardizing the dietary surveys^23^. However, our detailed standardization methods have previously been reported to allow for transparency^42-44^. The GDD was originally designed to estimate intakes of foods and nutrients with potential causal relationships with non-communicable diseases. As the data searches, extraction and harmonization did not include poultry, we are not able to estimate poultry intake, but we plan to do this in future iterations of the GDD^23^. Consequently, the omittance of poultry intake may underestimate total ASF consumption, particularly among children and adolescents in high-income countries, Latin America and the Caribbean, and China, where the per capita availability of poultry is highest^45^. Although absolute intake of poultry is likely to be lowest among children and adolescents in South Asia and sub-Saharan Africa^45^, poultry consumption may contribute a substantial proportion of total ASF among these populations. Additionally, we did not collect data on breastfeeding or formula use, and we were unable to account for energy intake from breastfeeding or formula among infants. Lastly, individual-level dietary surveys are subject to sampling and measurement bias, and despite incorporating additional uncertainty in the Bayesian hierarchical models, these types of bias cannot be ruled out^46^.

In conclusion, we found that global ASF consumption among children and adolescents was approximately 2 servings per day, but with substantial variation across regions, countries, age groups, parental education level, urbanicity and the types of ASF consumed.

## Methods

### Data sources and retrieval

We produced comprehensive, comparable estimates of dietary intakes of 53 major foods and nutrients in 185 countries as a part of the GDD. Detailed methods and standardized data collection have been reported^23,42-44,47^. In brief, we systematically searched for individual-level national surveys for dietary intakes worldwide, with additional data obtained through communication with researchers and government authorities^42^. We prioritized nationally and sub-nationally representative surveys, and surveys collected at the individual level using standardized 24-hour recalls, food frequency questionnaires or short standardized questionnaires (for example, Demographic Health Survey questionnaire)^42^. Surveys from large cohort studies or household budget surveys in populous countries were selected when nationally or sub-nationally representative individual-level surveys were not identified^42^. Surveys focused on special populations (for example, pregnant women, lactating women, individuals with a specific disease) were excluded^42^.

GDD 2018 incorporated 1,248 dietary surveys from 188 countries, comprising 99% of the world’s population^23^. Data on ASF consumption (milk, cheese, yogurt, eggs, seafood, unprocessed red meat, processed red meat) were reported in 498 surveys^23^, including 429 with data on children and adolescents, defined as between age 0 to 19 years (Supplementary Table 3). These 429 surveys included 3.3 million children from 125 countries, representing 93.1% of the global child population (Supplementary Table 4). Most surveys were nationally representative (88.1%), collected at the individual level (78.1%), and included data by rural and/or urban residence (71.1%) and by parental education (53.4%).

### Data extraction and harmonization

Data were extracted for each survey using standardized methods on survey characteristics and diet metrics, units, means and standard deviations of intake, in subgroups jointly stratified by age group, sex, parental education and urban/rural residence^23,42^. Standardized protocols assessed data for extraction errors and survey quality including selection bias, sample representativeness, response rate and validity of diet assessment method^23,42^. Data were standardized to mean individual intakes using the average of all days of dietary assessment; harmonized dietary definitions and units of measures across surveys; and adjusting for total energy based on age-specific energy intakes^23,42^. For children <1 year of age, intakes were energy adjusted to 700 kcal d^−1^; for 1–<2 years, 1,000 kcal d^−1^; for 2–5 years, 1,300 kcal d^−1^; for 6–10 years, 1,700 kcal d^−1^; and for 11–19 years, 2,000 kcal d^−1^ (refs. 23,42). Data harmonization and energy adjustment analyses were performed using SAS v9.4 (SAS Institute), Stata v14.0 (StataCorp LLC) and RStudio v1.1.453 (RStudio).

### Modelling and uncertainty

A Bayesian model was used to account for missingness, differences in survey methods, representativeness, time and uncertainty^23^. The model incorporated a nested hierarchical structure, with random effects by country and region, globally, and jointly stratified for age (<1, 1–2, 3–4, 5–9, 10–14, 15–19 years), sex (boys, girls), parental education (<6 years of education, ≥6 to <12 years, ≥12 years) and urbanicity (urban, rural residence)^23^. For each ASF, primary model inputs were stratified survey data on quantitative intakes, survey characteristics (dietary assessment method, diet metric) and country-year-specific covariates^23^. The model included overdispersion of survey-level variance for surveys that were not nationally representative or not stratified by smaller age groups (≤10 years), sex, education or urbanicity. Uncertainty of each stratum-specific estimate was quantified using 4,000 runs to determine posterior distributions of consumption jointly by country, year and demographic subgroup^23^. The median intake and 95% uncertainty interval for each stratum were calculated from the 50th, 2.5th and 97.5th percentiles of the 4,000 draws, respectively^23^. Validity was assessed by fivefold cross-validation (randomly omitting 20% of the raw survey data, run 5 times), comparing predicted versus observed intakes; and by assessment of implausible estimates and visual assessment of global heat maps^23^. A second time component Bayesian model was used to strengthen differences in estimates over time for diet factors with food or nutrient availability data (United Nations Food and Agriculture Organization Food Balance Sheets^45^ and the Global Expanded Nutrient Supply project^48^)^23^. The results were based on these two Bayesian models, as described in detail in the Supplementary Information. Analyses were completed using RStudio v3.3 and Stan v2.29.

### Statistical analysis

The model estimated mean intake of each ASF and its statistical uncertainty for each of the 72 population strata (jointly by age group, sex, education and urbanicity) from 185 countries, for 1990 and 2018. Using data on population weights, we estimated global, regional, national and within-country population subgroup intakes of ASF by calculating population-weighted averages of the stratum-specific estimates for each of the 72 demographic strata in each country-year^23^. Population weights for each stratum in 1990 and 2018 were derived from the United Nations Population Division^49^, with supplemental data on education and urbanicity from Barro–Lee^50^ and the United Nations^23,51^. Intakes were calculated as grams per day and servings per day or week using standardized portion sizes^23^. A serving size of milk was defined as 245 g; 42 g for cheese; 245 g for yogurt; 55 g for eggs; 100 g for seafood; 100 g for unprocessed red meat; and 50 g for total processed meat. Spearman correlations evaluated relationships between mean intakes of different ASF. When comparing subgroups and trends over time, absolute differences in consumption were calculated using all 4,000 posterior predictions at each stratum level to incorporate the full spectrum of uncertainty^23^. Differences in intakes between 1990 and 2018 were standardized to the 2018 population weights to account for changes in demographics over time^23^.

### Ethics statement

This modelling study was exempt from ethical review board approval because it was based on published data and nationally representative, de-identified data sets without personally identifiable information. Individual surveys underwent ethical review board approval required for the applicable local context.

## Extended Data

## Supplementary Material

Supplement

## Data Availability

The modelled estimates are available for download from the Global Dietary Database (https://www.globaldietarydatabase.org/). Survey-level information and original data download weblinks are also provided for all public surveys; and survey-level microdata or stratum-level aggregate data are provided for direct download for all non-public surveys granted consent for public sharing by the data owner. Data on national food and nutrient supplies are available for download from the United Nations Food and Agriculture Organization (https://www.fao.org/faostat/en/#data) and the Global Expanded Nutrient Supply (GENuS) model dataset (https://dataverse.harvard.edu/dataverse/GENuS). Data on population demographics are available for download from the United Nations Population Division (https://population.un.org/wpp/DataQuery/).

## Code availability

The statistical coding is available from the corresponding author on reasonable request.

## Global Dietary Database

Murat Bas^6^, Jemal Haidar Ali^7^, Suhad Abumweis^8^, Anand Krishnan^9^, Puneet Misra^9^, Nahla Chawkat Hwalla^10^, Chandrashekar Janakiram^11^, Nur Indrawaty Liputo^12^, Abdulrahman Musaiger^13^, Farhad Pourfarzi^14^, Iftikhar Alam^15^, Karin DeRidder^16^, Celine Termote^17^, Anjum Memon^18^, Aida Turrini^19^, Elisabetta Lupotto^19^, Raffaela Piccinelli^19^, Stefania Sette^19^, Karim Anzid^20^, Marieke Vossenaar^21^, Paramita Mazumdar^22^, Ingrid Rached^23^, Alicia Rovirosa^24^, María Elisa Zapata^24^, Tamene Taye Asayehu^25^, Francis Oduor^26^, Julia Boedecker^26^, Lilian Aluso^26^, Johana Ortiz-Ulloa^27^, J. V. Meenakshi^28^, Michelle Castro^29^, Giuseppe Grosso^30^, Anna Waskiewicz^31^, Umber S. Khan^32^, Anastasia Thanopoulou^33^, Reza Malekzadeh^34^, Neville Calleja^35^, Marga Ocke^36^, Zohreh Etemad^36^, Mohannad Al Nsour^37^, Lydiah M. Waswa^38^, Eha Nurk^39^, Joanne Arsenault^40^, Patricio Lopez-Jaramillo^41^, Abla Mehio Sibai^42^, Albertino Damasceno^43^, Carukshi Arambepola^44^, Carla Lopes^45^, Milton Severo^45^, Nuno Lunet^45^, Duarte Torres^46^, Heli Tapanainen^47^, Jaana Lindstrom^47^, Suvi Virtanen^47^, Cristina Palacios^48^, Eva Roos^49^, Imelda Angeles Agdeppa^50^, Josie Desnacido^50^, Mario Capanzana^51^, Anoop Misra^52^, Ilse Khouw^53^, Swee Ai Ng^53^, Edna Gamboa Delgado^54^, Mauricio Caballero^55^, Johanna Otero^56^, Hae-Jeung Lee^57^, Eda Koksal^58^, Idris Guessous^59^, Carl Lachat^60^, Stefaan De Henauw^60^, Ali Reza Rahbar^61^, Alison Tedstone^61^, Androniki Naska^61^, Angie Mathee^61^, Annie Ling^61^, Bemnet Tedla^61^, Beth Hopping^61^, Brahmam Ginnela^61^, Catherine Leclercq^61^, Charmaine Duante^61^, Christian Haerpfer^61^, Christine Hotz^61^, Christos Pitsavos^61^, Colin Rehm^61^, Coline van Oosterhout^61^, Corazon Cerdena^61^, Debbie Bradshaw^61^, Dimitrios Trichopoulos^61^, Dorothy Gauci^61^, Dulitha Fernando^61^, Elzbieta Sygnowska^61^, Erkki Vartiainen^61^, Farshad Farzadfar^61^, Gabor Zajkas^61^, Gillian Swan^61^, Guansheng Ma^61^, Gulden Pekcan^61^, Hajah Masni Ibrahim^61^, Harri Sinkko^61^, Helene Enghardt Barbieri^61^, Isabelle Sioen^61^, Jannicke Myhre^61^, Jean-Michel Gaspoz^61^, Jillian Odenkirk^61^, Kanitta Bundhamcharoen^61^, Keiu Nelis^61^, Khairul Zarina^61^, Lajos Biro^61^, Lars Johansson^61^, Laufey Steingrimsdottir^61^, Leanne Riley^61^, Mabel Yap^61^, Manami Inoue^61^, Maria Szabo^61^, Marja-Leena Ovaskainen^61^, Meei-Shyuan Lee^61^, Mei Fen Chan^61^, Melanie Cowan^61^, Mirnalini Kandiah^61^, Ola Kally^61^, Olof Jonsdottir^61^, Pam Palmer^61^, Peter Vollenweider^61^, Philippos Orfanos^61^, Renzo Asciak^61^, Robert Templeton^61^, Rokiah Don^61^, Roseyati Yaakub^61^, Rusidah Selamat^61^, Safiah Yusof^61^, Sameer Al-Zenki^61^, Shu-Yi Hung^61^, Sigrid Beer-Borst^61^, Suh Wu^61^, Widjaja Lukito^61^, Wilbur Hadden^61^, Wulf Becker^61^, Xia Cao^61^, Yi Ma^61^, Yuen Lai^61^, Zaiton Hjdaud^61^, Jennifer Ali^62^, Ron Gravel^62^, Tina Tao^62^, Jacob Lennert Veerman^63^, Shashi Chiplonkar^64^, Mustafa Arici^65^, Le Tran Ngoan^66^, Demosthenes Panagiotakos^67^, Yanping Li^68^, Antonia Trichopoulou^69^, Noel Barengo^70^, Anuradha Khadilkar^71^, Veena Ekbote^71^, Noushin Mohammadifard^72^, Irina Kovalskys^73^, Avula Laxmaiah^74^, Harikumar Rachakulla^74^, Hemalatha Rajkumar^74^, Indrapal Meshram^74^, Laxmaiah Avula^74^, Nimmathota Arlappa^74^, Rajkumar Hemalatha^74^, Licia lacoviello^75^,^76^, Marialaura Bonaccio^75^, Simona Costanzo^75^, Yves Martin-Prevel^77^, Katia Castetbon^78^, Nattinee Jitnarin^79^, Yao-Te Hsieh^80^, Sonia Olivares^81^, Gabriela Tejeda^82^, Aida Hadziomeragic^83^, Amanda de Moura Souza^84^, Wen-Harn Pan^85^, Inge Huybrechts^86^, Alan de Brauw^87^, Mourad Moursi^87^, Maryam Maghroun^88^, Augustin Nawidimbasba Zeba^89^, Nizal Sarrafzadegan^90^, Lital Keinan-Boker^91^, Rebecca Goldsmith^91^, Tal Shimony^91^, Irmgard Jordan^92^, Shivanand C. Mastiholi^93^, Moses Mwangi^94^, Yeri Kombe^94^, Zipporah Bukania^94^, Eman Alissa^95^, Nasser Al-Daghri^96^, Shaun Sabico^96^, Martin Gulliford^97^, Tshilenge S. Diba^98^, Kyungwon Oh^99^, Sanghui Kweon^99^, Sihyun Park^99^, Yoonsu Cho^100^, Suad Al-Hooti^101^, Chanthaly Luangphaxay^102^, Daovieng Douangvichit^102^, Latsamy Siengsounthone^102^, Pedro Marques-Vidal^103^, Constance Rybak^104^, Amy Luke^105^, Noppawan Piaseu^106^, Nipa Rojroongwasinkul^107^, Kalyana Sundram^108^, Donka Baykova^109^, Parvin Abedi^110^, Sandjaja Sandjaja^111^, Fariza Fadzil^112^, Noriklil Bukhary Ismail Bukhary^113^, Pascal Bovet^114,115^, Yu Chen^116^, Norie Sawada^117^, Shoichiro Tsugane^117^, Lalka Rangelova^118^, Stefka Petrova^118^, Vesselka Duleva^118^, Anna Karin Lindroos^119^, Jessica Petrelius Sipinen^119^, Lotta Moraeus^119^, Per Bergman^119^, Ward Siamusantu^120^, Lucjan Szponar^121^, Hsing-Yi Chang^122^, Makiko Sekiyama^123^, Khanh Le Nguyen Bao^124^, Balakrishna Nagalla^125^, Kalpagam Polasa^125^, Sesikeran Boindala^125^, Jalila El Ati^126^, Ivonne Ramirez Silva^127^, Juan Rivera Dommarco^127^, Simon Barquera^127^, Sonia Rodríguez Ramírez^127^, Daniel Illescas-Zarate^128^, Luz Maria Sanchez-Romero^128^, Nayu Ikeda^129^, Sahar Zaghloul^130^, Anahita Houshiar-rad^131^, Fatemeh Mohammadi-Nasrabadi^131^, Morteza Abdollahi^131^, Khun-Aik Chuah^132^, Zaleha Abdullah Mahdy^132^, Alison Eldridge^133^, Eric L. Ding^134^, Herculina Kruger^135^, Sigrun Henjum^136^, Anne Fernandez^137^, Milton Fabian Suarez-Ortegon^138^, Nawal Al Hamad^139^, Veronika Janská^140^, Reema Tayyem^141^, Parvin Mirmiran^142^, Roya Kelishadi^143^, Eva Warensjo Lemming^144^, Almut Richter^145^, Gert Mensink^145^, Lothar Wieler^145^, Daniel Hoffman^146^, Benoit Salanave^147^, Cho-il Kim^148^, Rebecca Kuriyan-Raj^149^, Sumathi Swaminathan^149^, Didier Garriguet^150^, Saeed Dastgiri^151^, Sirje Vaask^152^, Tilakavati Karupaiah^153^, Fatemeh Vida Zohoori^154^, Alireza Esteghamati^155^, Maryam Hashemian^156,157^, Sina Noshad^155^, Elizabeth Mwaniki^156^, Elizabeth Yakes-Jimenez^158^, Justin Chileshe^159^, Sydney Mwanza^159^, Lydia Lera Marques^160^, Alan Martin Preston^161^, Samuel Duran Aguero^162^, Mariana Oleas^163^, Luz Posada^164^, Angelica Ochoa^165^, Khadijah Shamsuddin^166^, Zalilah Mohd Shariff^167^, Hamid Jan Bin Jan Mohamed^168^, Wan Manan^168^, Anca Nicolau^169^, Cornelia Tudorie^169^, Bee Koon Poh^170^, Pamela Abbott^171^, Mohammadreza Pakseresht^172^, Sangita Sharma^172^, Tor Strand^173^, Ute Alexy^174^, Ute Nöthlings^174^, Jan Carmikle^174^, Ken Brown^175^, Jeremy Koster^176^, Indu Waidyatilaka^177^, Pulani Lanerolle^177^, Ranil Jayawardena^177^, Julie M. Long^178^, K. Michael Hambidge^178^, Nancy F. Krebs^178^, Aminul Haque^179^, Gudrun B. Keding^180^, Liisa Korkalo^181^, Maijaliisa Erkkola^181^, Riitta Freese^181^, Laila Eleraky^182^, Wolfgang Stuetz^182^, Inga Thorsdottir^183^, Ingibjorg Gunnarsdottir^183^, Lluis Serra-Majem^184^, Foong Ming Moy^185^, Simon Anderson^186^, Rajesh Jeewon^187^, Corina Aurelia Zugravu^188^, Linda Adair^189^, Shu Wen Ng^189^, Sheila Skeaff^190^, Dirce Marchioni^191^, Regina Fisberg^191^, Carol Henry^192^, Getahun Ersino^192^, Gordon Zello^192^, Alexa Meyer^193^, Ibrahim Elmadfa^193^, Claudette Mitchell^194^, David Balfour^194^, Johanna M. Geleijnse^195^, Mark Manary^196^, Tatyana El-kour^197^, Laetitia Nikiema^198^, Masoud Mirzaei^199^ & Rubina Hakeem^200^

^6^Acibadem University, Istanbul, Turkey. ^7^Addis Ababa University, Addis Ababa, Ethiopia. ^8^Al Ain University, Abu Dhabi, UAE. ^9^All India Institute of Medical Sciences, New Delhi, India. ^10^American University of Beirut, Beirut, Lebanon. ^11^Amrita School of Dentistry, Eranakulum, India. ^12^Andalas University, Padang, Indonesia. ^13^Arab Center for Nutrition, Manama, Bahrain. ^14^Ardabil University of Medical Sciences, Ardabil, Iran. ^15^Bacha Khan University, Charsadda, Pakistan. ^16^Belgian Public Health Institute, Brussels, Belgium. ^17^Biodiversity International, Maccarese, Italy. ^18^Brighton and Sussex Medical School, Brighton, UK. ^19^CREA-Alimenti e Nutrizione, Rome, Italy. ^20^Cadi Ayyad University, Benguerir, Morocco. ^21^Center for Studies of Sensory Impairment, Aging and Metabolism (CeSSIAM), Guatemala City, Guatemala. ^22^Centre For Media Studies, New Delhi, India. ^23^Centro de Atencion Nutricional Antimano (CANIA), Miami, FL, USA. ^24^Centro de Estudios sobre Nutrición Infantil (CESNI), Buenos Aires, Argentina. ^25^College of Applied Sciences, Department of Food Science and Applied Nutrition, Addis Ababa Science and Technology University, Addis Ababa, Ethiopia. ^26^Consultative Group on International Agricultural Research (CGIAR), Montpellier, France. ^27^Cuenca University, Cuenca, Ecuador. ^28^Delhi School of Economics, University of Delhi, Delhi, India. ^29^Departamento de Alimentacao Escolar, Sao Paulo, Brazil. ^30^Department of Biomedical and Biotechnological Sciences, University of Catania, Catania, Italy. ^31^Department of CVD Epidemiology, Prevention and Health Promotion, Institute of Cardiology, Warsaw, Poland. ^32^Department of Community Health Sciences, Aga Khan University, Karachi, Pakistan. ^33^Diabetes Center, Second Department of Internal Medicine, Athens University, Athens, Greece. ^34^Digestive Disease Research Institute, Tehran University of Medical Sciences, Tehran, Iran. ^35^Directorate for Health Information & Research, Tarxien, Malta. ^36^Dutch National Institute for Public Health and the Environment (RIVM), Bilthoven, Netherlands. ^37^Eastern Mediterranean Public Health Network (EMPHNET), Amman, Jordan. ^38^Egerton University, Njoro, Kenya. ^39^Estonian National Institute for Health Development, Tallinn, Estonia. ^40^FHI Solutions, Washington, DC, USA. ^41^FOSCAL and UDES, Bucaramanga, Colombia. ^42^Faculty of Health Sciences, American University of Beirut, Beirut, Lebanon. ^43^Faculty of Medicine, Eduardo Mondlane University, Maputo, Mozambique. ^44^Faculty of Medicine, University of Colombo, Colombo, Sri Lanka. ^45^Faculty of Medicine/Institute of Public Health, University of Porto, Porto, Portugal. ^46^Faculty of Nutrition and Food Sciences, University of Porto, Porto, Portugal. ^47^Finnish Institute for Health and Welfare, Helsinki, Finland. ^48^Florida International University, Miami, FL, USA. ^49^Folkhälsan Research Center, Helsinki, Finland. ^50^Food and Nutrition Research Institue (DOST-FNRI), Manila, Philippines. ^51^Food and Nutrition Research Institute, Department of Science and Technology, Taguig City, Philippines. ^52^Fortis CDOC Center for Excellence for Diabetes, New Delhi, India. ^53^FrieslandCampina, Amersfoort, The Netherlands. ^54^Fundacion Cardiovascular de Colombia, Bucaramanga, Colombia. ^55^Fundacion INFANT and Consejo Nacional De Investigaciones Cientificas y Tecnicas (CONICET), Buenos Aires, Argentina. ^56^Fundacion Oftalmologica de Santander (FOSCAL), Floridablanca, Colombia. ^57^Gachon University, Seongnam-si, South Korea. ^58^Gazi University, Yenimahalle/Ankara, Turkey. ^59^Geneva University Hospitals, Geneva, Switzerland. ^60^Ghent University, Ghent, Belgium. ^61^Global Dietary Database Consortium, Boston, MA, USA. ^62^Government of Canada, Statistics Canada, Ottawa, Canada. ^63^Griffith University, Gold Coast, Queensland, Australia. ^64^HC Jehangir Medical Research Institute, Pune, India. ^65^Hacettepe University Faculty of Medicine, Ankara, Turkey. ^66^Hanoi Medical University, Hanoi, Vietnam. ^67^Harokopio University, Athens (Kallithea), Greece. ^68^Harvard School of Public Health, Cambridge, MA, USA. ^69^Hellenic Health Foundation and University of Athens, Athens, Greece. ^70^Herbert Wetheim College of Medicine, Miami, FL, USA. ^71^Hirabai Cowasji Jehangir Medical Research Institute, Pune, India. ^72^Hypertension Research Center, Cardiovascular Research Center, Isfahan University of Medical Sciences, Isfahan, Iran. ^73^ICCAS (Instituto para la Cooperacion Científica en Ambiente y Salud), Buenos Aires, Argentina. ^74^ICMR-National Institute of Nutrition, Hyderabad, India. ^75^IRCCS Neuromed, Pozzilli, Italy. ^76^University of Insubria, Varese, Italy. ^77^Institut de Recherche pour le Developpement, Montepellier, France. ^78^Institut de Veille Sanitaire, Bobigny, France. ^79^Institute for International Investigation, NDRI-USA, New York, NY, USA. ^80^Institute of Biomedical Sciences, Academia Sinica, Taipei, Taiwan. ^81^Institute of Nutrition and Food Technology (INTA), University of Chile, Santiago, Chile. ^82^Institute of Nutrition in Central America and Panama (INCAP), Guatemala City, Guatemala. ^83^Institute of Public Health of Federation of Bosnia and Herzegovina, Sarajevo, Bosnia and Herzegovina. ^84^Institute of Studies in Public Health, Federal University of Rio de Janeiro (UFRJ), Rio de Janeiro, Brazil. ^85^Institutes of Biomedical Sciences, Academia Sinica, Taipei, Taiwan. ^86^International Agency for Research on Cancer, Lyon, France. ^87^International Food Policy Research Institute (IFPRI), Washington, DC, USA. ^88^Interventional Cardiology Research Center, Cardiovascular Research Center, Isfahan University of Medical Sciences, Isfahan, Iran. ^89^Intitut de Recherche en Sciences de la Sante, Bobo Dioulasso, Burkina Faso. ^90^Isfahan Cardiovascular Research Center, Cardiovascular Research Center, Isfahan University of Medical Sciences, Isfahan, Iran. ^91^Israel Center for Disease Control, Tel-Hashomer, Israel. ^92^Justus Liebig University Giessen, Giessen, Germany. ^93^Jawaharlal Nehru Medical College, Belagavi, India. ^94^Kenya Medical Research Institute, Nairobi, Kenya. ^95^King Abdulaziz University, Jeddah, Saudi Arabia. ^96^King Saud University, Riyadh, Saudi Arabia. ^97^King’s College London, London, UK. ^98^Kinshasa School of Public Health, Kinshasa, Democratic Republic of Congo. ^99^Korea Disease Control and Prevention Agency (KDCA), Cheongju, Korea. ^100^Korea University, Seoul, Korea. ^101^Kuwait Institute for Scientific Research, Safat, Kuwait. ^102^Lao Tropical and Public Health Institute, Vientiane Capital, Lao PDR. ^103^Lausanne University Hospital (CHUV) and University of Lausanne, Lausanne, Switzerland. ^104^Leibniz Centre for Agricultural Landscape Research, Muncheberg, Germany. ^105^Loyola University Chicago, Chicago, IL, USA. ^106^Mahidol University, Bangkok, Thailand. ^107^Mahidol University, Pathom, Thailand. ^108^Malaysian Palm Oil Council (MPOC), Kelana Jaya, Malaysia. ^109^Medical Center Markovs, Sofia, Bulgaria. ^110^Menopause Andropause Research Center, Ahvaz Jundishapur University of Medical Sciences, Ahvaz, Iran. ^111^Ministry of Health, Jakarta, Indonesia. ^112^Ministry of Health, Kuala Lumpur, Malaysia. ^113^Ministry of Health, Sungai Besar, Malaysia. ^114^Ministry of Health, Victoria, Seychelles. ^115^University Center for Primary Care and Public Health (Unisanté), Lausanne, Switzerland. ^116^NYU School of Medicine, New York, NY, USA. ^117^National Cancer Center Institute for Cancer Control, Tokyo, Japan. ^118^National Centre of Public Health and Analyses (NCPHA), Sofia, Bulgaria. ^119^National Food Agency, Uppsala, Sweden. ^120^National Food and Nutrition Commission, Lusaka, Zambia. ^121^National Food and Nutrition Institute, Warsaw, Poland. ^122^National Health Research Institutes, Zhunan Township, Taiwan. ^123^Health and Environmental Risk Division, National Institute for Environmental Studies, Tsukuba, Japan. ^124^National Institute of Nutrition, Hanoi, Vietnam. ^125^National Institute of Nutrition, Hyderabad, India. ^126^National Institute of Nutrition and Food Technology & SURVEN RL, Tunis, Tunisia. ^127^National Institute of Public Health (INSP), Cuernavaca, Mexico. ^128^National Institute of Public Health (INSP), Mexico City, Mexico. ^129^National Institutes of Biomedical Innovation, Health and Nutrition, Tokyo, Japan. ^130^National Nutrition Institute, Cairo, Egypt. ^131^National Nutrition and Food Technology Research Institute (NNFTRI): SBMU, Tehran, Iran. ^132^National University of Malaysia (UKM), Kuala Lumpur, Malaysia. ^133^Nestlé Research, Lausanne, Switzerland. ^134^New England Complex Systems Institute, Cambridge, MA, USA. ^135^North-West University, Potchefstroom, South Africa. ^136^Oslo Metropolitan University (OsloMet), Oslo, Norway. ^137^Perdana University–Royal College of Surgeons in Ireland, Puchong, Malaysia. ^138^Pontificia Universidad Javeriana Seccional, Cali, Colombia. ^139^Public Authority For Food and Nutrition, Sabah Al Salem, Kuwait. ^140^Public Health Authority of the Slovak Republic, Bratislava, Slovak Republic. ^141^Qatar University and University of Jordan, Doha, Qatar. ^142^Research Institute for Endocrine Sciences, Shahid Beheshti University of Medical Sciences, Tehran, Iran. ^143^Research Institute for Primordial Prevention of NCD, Isfahan University of Medical Sciences, Isfahan, Iran. ^144^Risk and Benefit Assessment Department, Swedish Food Agency, Uppsala, Sweden. ^145^Robert Koch Institute, Berlin, Germany. ^146^Rutgers University, New Brunswick, NJ, USA. ^147^Santé publique France, Saint Maurice, France. ^148^Seoul National University, Seoul, Korea. ^149^St John’s Research Institute, Bangalore, India. ^150^Statistics Canada, Ottawa, Ontario, Canada. ^151^Tabriz University of Medical Sciences, Tabriz, Iran. ^152^Tallinn University, Tallinn, Estonia. ^153^Taylor’s University, Selangor, Malaysia. ^154^Teesside University, Middlesbrough, UK. ^155^Tehran University of Medical Sciences, Tehran, Iran. ^156^The Technical University of Kenya, Nairobi, Kenya. ^157^Utica University, Tehran, Iran. ^158^The University of New Mexico, Albuquerque, NM, USA. ^159^Tropical Diseases Research Centre, Ndola, Zambia. ^160^Unidad de Nutricion Publica, Macul, Chile. ^161^Department of Biochemistry, Medical Sciences, University of Puerto Rico, San Juan, Puerto Rico. ^162^Universidad San Sebastian, Providencia, Chile. ^163^Universidad Tecnica del Norte, Ibarra, Ecuador. ^164^Universidad de Antioquia, Medellin, Colombia. ^165^Universidad de Cuenca, Cuenca, Ecuador. ^166^Universiti Kebangsaan Malaysia Medical Centre, Kuala Lumpur, Malaysia. ^167^Universiti Putra Malaysia, Serdang, Malaysia. ^168^Universiti Sains Malaysia, Kubang Kerian, Malaysia. ^169^University Dunarea de Jos, Galati, Romania. ^170^University Kebangsaan Malaysia, Selangor, Malaysia. ^171^University of Aberdeen, Aberdeen, UK. ^172^University of Alberta, Edmonton, Alberta, Canada. ^173^University of Bergen, Bergen, Norway. ^174^Department of Nutrition and Food Sciences, University of Bonn, Bonn, Germany. ^175^University of California Davis, Davis, CA, USA. ^176^University of Cincinnati, Cincinnati, OH, USA. ^177^University of Colombo, Colombo, Sri Lanka. ^178^University of Colorado School of Medicine, Aurora, CO, USA. ^179^University of Dhaka, Dhaka, Bangladesh. ^180^University of Goettingen, Goettingen, Germany. ^181^Department of Food and Nutrition, University of Helsinki, Helsinki, Finland. ^182^University of Hohenheim, Stuttgart, Germany. ^183^University of Iceland, Reykjavík, Iceland. ^184^University of Las Palmas de Gran Canaria (ULPGC), Canary Islands, Las Palmas, Spain. ^185^University of Malaya, Kuala Lumpur, Malaysia. ^186^University of Manchester, Manchester, UK. ^187^University of Mauritius, Reduit, Mauritius. ^188^University of Medicine and Pharmacy Carol Davila, Bucharest, Romania. ^189^University of North Carolina at Chapel Hill, Chapel Hill, NC, USA. ^190^University of Otago, Dunedin, New Zealand. ^191^University of Sao Paulo, Sao Paulo, Brazil. ^192^University of Saskatchewan, Saskatoon, Saskatchewan, Canada. ^193^University of Vienna, Vienna, Austria. ^194^University of the Southern Caribbean, Port-of-Spain, Trinidad and Tobago. ^195^Wageningen University, Wageningen, Netherlands. ^196^Washington University in St. Louis, St. Louis, MO, USA. ^197^World Health Organization (WHO), Amman, Jordan. ^198^World Health Organization (WHO), Geneva, Switzerland. ^199^Yazd Cardiovascular Research Centre, Shahid Sadoughi University of Medical Sciences, Yazd, Iran. ^200^Ziauddin University Karachi, Karachi City, Pakistan.
